# Brain DNA methylomic analysis of frontotemporal lobar degeneration reveals *OTUD4* in shared dysregulated signatures across pathological subtypes

**DOI:** 10.1007/s00401-023-02583-z

**Published:** 2023-05-07

**Authors:** Katherine Fodder, Megha Murthy, Patrizia Rizzu, Christina E. Toomey, Rahat Hasan, Jack Humphrey, Towfique Raj, Katie Lunnon, Jonathan Mill, Peter Heutink, Tammaryn Lashley, Conceição Bettencourt

**Affiliations:** 1 Queen Square Brain Bank for Neurological Disorders, UCL Queen Square Institute of Neurology, London, UK; 2 Department of Neurodegenerative Disease, UCL Queen Square Institute of Neurology, London, UK; 3 Department of Clinical and Movement Neurosciences, UCL Queen Square Institute of Neurology, London, UK; 4 German Center for Neurodegenerative Diseases (DZNE), Tübingen, Germany; 5 The Francis Crick Institute, London, UK; 6 Nash Family Department of Neuroscience and Friedman Brain Institute, Icahn School of Medicine at Mount Sinai, New York, NY USA; 7 Department of Clinical and Biomedical Sciences, Faculty of Health and Life Sciences, University of Exeter, Exeter, UK; 8 Alector, Inc., South San Francisco, CA USA

**Keywords:** DNA methylation, Frontotemporal dementia, Progressive supranuclear palsy, Human brain tissue, EWAS, Co-methylation

## Abstract

Frontotemporal lobar degeneration (FTLD) is an umbrella term describing the neuropathology of a clinically, genetically and pathologically heterogeneous group of diseases, including frontotemporal dementia (FTD) and progressive supranuclear palsy (PSP). Among the major FTLD pathological subgroups, FTLD with TDP-43 positive inclusions (FTLD-TDP) and FTLD with tau-positive inclusions (FTLD-tau) are the most common, representing about 90% of the cases. Although alterations in DNA methylation have been consistently associated with neurodegenerative diseases, including Alzheimer’s disease and Parkinson’s disease, little is known for FTLD and its heterogeneous subgroups and subtypes. The main goal of this study was to investigate DNA methylation variation in FTLD-TDP and FTLD-tau. We used frontal cortex genome-wide DNA methylation profiles from three FTLD cohorts (142 FTLD cases and 92 controls), generated using the Illumina 450K or EPIC microarrays. We performed epigenome-wide association studies (EWAS) for each cohort followed by meta-analysis to identify shared differentially methylated loci across FTLD subgroups/subtypes. In addition, we used weighted gene correlation network analysis to identify co-methylation signatures associated with FTLD and other disease-related traits. Wherever possible, we also incorporated relevant gene/protein expression data. After accounting for a conservative Bonferroni multiple testing correction, the EWAS meta-analysis revealed two differentially methylated loci in FTLD, one annotated to *OTUD4* (5’UTR-shore) and the other to *NFATC1* (gene body-island)*.* Of these loci, *OTUD4* showed consistent upregulation of mRNA and protein expression in FTLD. In addition, in the three independent co-methylation networks, *OTUD4*-containing modules were enriched for EWAS meta-analysis top loci and were strongly associated with the FTLD status. These co-methylation modules were enriched for genes implicated in the ubiquitin system, RNA/stress granule formation and glutamatergic synaptic signalling. Altogether, our findings identified novel FTLD-associated loci, and support a role for DNA methylation as a mechanism involved in the dysregulation of biological processes relevant to FTLD, highlighting novel potential avenues for therapeutic development.

**Supplementary Information:**

The online version contains supplementary material available at 10.1007/s00401-023-02583-z.

## Introduction

Frontotemporal lobar degeneration (FTLD) is an umbrella term describing the neuropathology of a group of neurodegenerative disorders, which are characterised by the selective degeneration of the frontal and temporal lobes of the brain. These disorders are clinically, pathologically and genetically heterogeneous. Clinically, patients with FTLD frequently present with frontotemporal dementia (FTD), which is the second most common form of early onset dementia and is often associated with behavioural and language changes. A fraction of patients may present with or develop Parkinsonism as part of their disease, including those with progressive supranuclear palsy (PSP), and frontotemporal dementia and parkinsonism linked to chromosome 17 (FTDP-17). An overlap with amyotrophic lateral sclerosis/motor neuron disease (ALS/MND) is also observed in a proportion of patients with FTLD, highlighting a spectrum of clinical phenotypes that relate to shared neuropathologic features [17, 42].

A considerable number of FTLD cases report a positive family history (30–50%), and the majority of familial cases can be attributed to mutations in three genes, namely chromosome 9 open reading frame 72 (*C9orf72*), progranulin (*GRN*), and microtubule-associated protein tau (*MAPT*). Apart from those cases in which a genetic mutation has been identified, neuropathological assessment is essential to confirm the disease entity underlying FTLD. The neuropathological classification of FTLDs, based on the presence/absence of specific proteinaceous inclusions, recognises five major subgroups. FTLD with 43 kDa transactive response DNA-binding protein (TDP-43) positive inclusions (FTLD-TDP), and with tau-positive inclusions (FTLD-tau), account for the vast majority of cases, representing around 50% and 40% of FTLD cases, respectively [28, 60].

Even though progress has been made in identifying genetic risk factors for diseases under the FTLD umbrella [19, 24, 35, 67, 88], the molecular mechanisms driving FTLD pathology are not completely understood. Mounting evidence reveals changes in the FTLD brain transcriptional landscapes [3, 15, 29, 31, 82]. However, studies investigating non-sequence-based regulatory mechanisms such as epigenetic modifications in FTLD brain tissue are limited [9, 52, 84, 87]. Variable DNA methylation, the most well-studied epigenetic modification, has consistently been associated with Alzheimer’s disease pathology in epigenome-wide studies (EWAS) and subsequent meta-analyses [73, 75, 90]. In FTLD, brain tissue EWAS are scarce and limited to a single PSP study [84].

To investigate further the relevance of DNA methylation variation in FTLD, we set out a study investigating epigenome-wide DNA methylation variation in frontal lobe tissue from three cohorts, spanning different subtypes of FTLD-TDP and FTLD-tau subgroups, followed by an EWAS meta-analysis, co-methylation network analysis in each cohort, and subsequent module preservation analysis in the other datasets. Through the EWAS meta-analysis we identified two differentially methylated loci shared across the FTLD subgroups and subtypes after a conservative Bonferroni correction for multiple testing. These methylation sites were annotated to *OTUD4* (5’UTR-shore) and *NFATC1* (gene body-island). We also identified co-methylation modules associated with the FTLD status, FTLD subtypes, and pathological features (e.g. brain atrophy and severity of neuronal loss). Functional and cellular enrichment analyses have shown an overrepresentation of gene ontology terms related to regulation of gene expression and the ubiquitin system as well as specific cell types, including pyramidal neurons and endothelial cells, across FTLD subgroups and subtypes. In all three independent co-methylation networks, *OTUD4*-containing modules were enriched for top EWAS meta-analysis loci, and were strongly associated with the disease status, further supporting their role in FTLD. Our findings implicate DNA methylation in the dysregulation of important processes in FTLD, including the ubiquitin system, RNA/stress granule formation and glutamatergic synaptic signalling.

## Methods

### Demographic and clinical characteristics of post-mortem brain donors

**Fig. 1:**
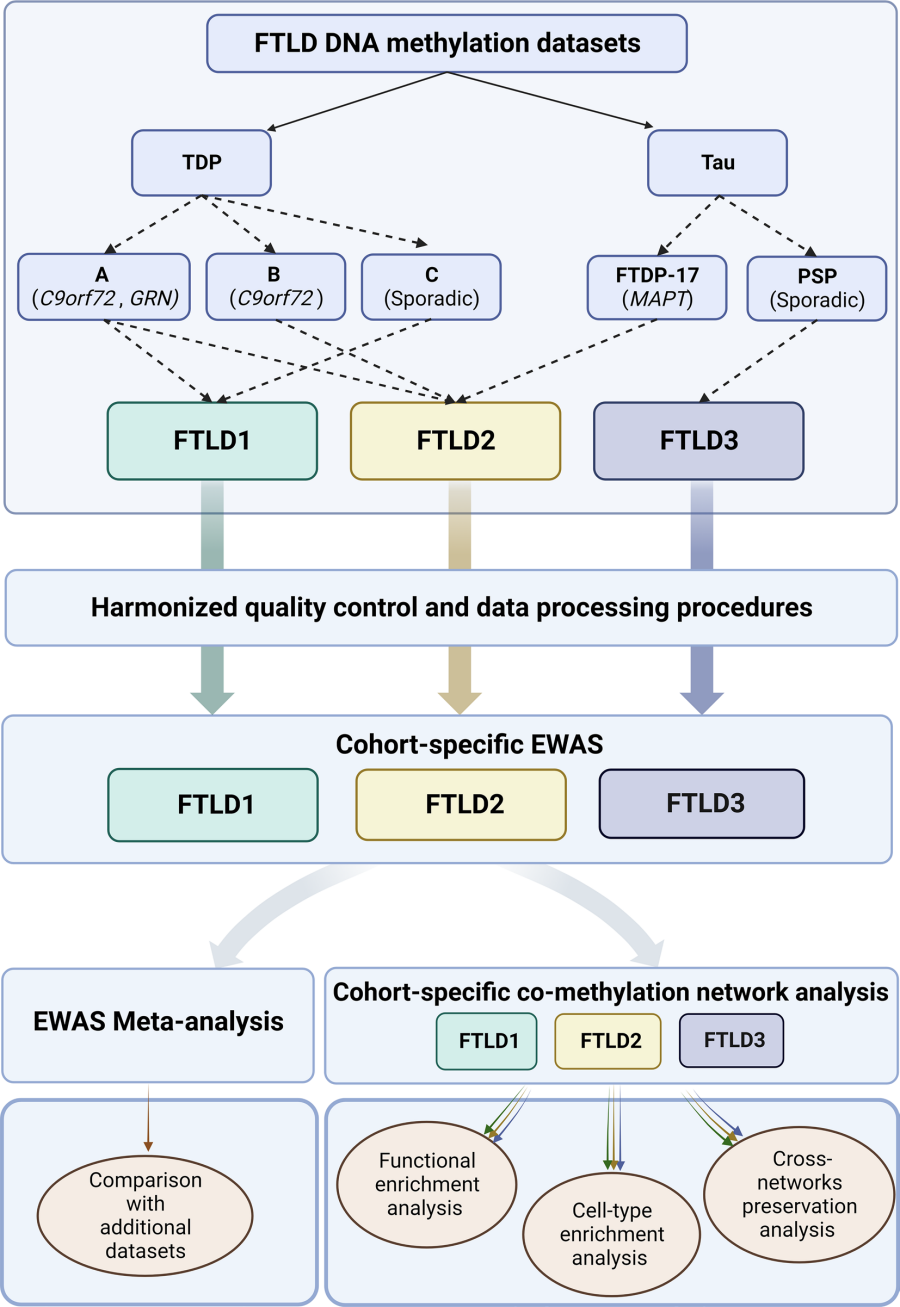
Outline of the study design and analysis framework. FTLD, Frontotemporal lobar degeneration; PSP, Progressive supranuclear palsy. Figure created with BioRender

For FTLD cohort 1 (FTLD1, *N* = 23), all post-mortem tissues originated from brains donated to the Queen Square Brain Bank archives, where tissues are stored under a licence from the Human Tissue authority (No. 12198). Both the brain donation programme and protocols have received ethical approval for donation and research by the NRES Committee London—Central. All cases were characterised by age, gender, disease history (including disease onset and duration) as well as neuropathological findings. For FTLD cohort 2 (FTLD2, *N* = 48), all post-mortem tissues were obtained under a Material Transfer Agreement from the Netherlands Brain Bank, and MRC Kings College London, as described by Menden et al. [54]. For FTLD cohort 3 (FTLD3, *N* = 163, after quality control), data made available by Weber et al. [84] was retrieved from GEO (accession code GSE75704). Figure 1 shows an outline of the study design and analysis framework. More details on each cohort are presented in Table 1.

**Table 1:** Pathological and demographic characteristics of the three FTLD cohorts and selected models for cohort-specific EWAS

Cohort	Pathological FTLD subtypes and controls included after quality control	Mean age ± SD (years)	Sex	Regression models used for cohort-specific EWAS
FTLD1	FTLD (*N* = 15)	70.07 ± 5.59	7M/8F	~ *0* + *disease* + *age* + *sex* + *SOX10*^+^ *proportions* + *Double*^*−*^* proportions* + *array* (0 surrogate variables detected)
	FTLD-TDP type A (*C9orf72* mutation carriers, *N* = 7)	66.86 ± 4.85	3M/4F	
	FTLD-TDP type C (sporadic, *N* = 8)	72.88 ± 4.79	4M/4F	
	Controls (*N* = 8)	75.75 ± 5.63	3M/5F	
FTLD2 [54]	FTLD (*N* = 34)	63.18 ± 7.92	14M/20F	~ *0* + *disease* + *age* + *sex* + *SOX10*^+^ *proportions* + *Double*^*−*^* proportions* + *array* + *slide* (0 surrogate variables detected)
	FTLD-TDP type A (*GRN* mutation carriers, *N* = 7)	65.57 ± 7.63	2M/5F	
	FTLD-TDP type B (*C9orf72* mutation carriers, *N* = 14)	64.57 ± 8.41	5M/9F	
	FTLD-tau (*MAPT* mutation carriers, *N* = 13)	60.92 ± 7.60	7M/6F	
	Controls (*N* = 14)	78.43 ± 11.76	5M/9F	
FTLD3 [84]	FTLD (*N* = 93)	71.16 ± 5.32	54M/39F	~ *0* + *disease* + *age* + *sex* + *SOX10*^+^ *proportions* + *Double*^*−*^* proportions* + *array* + *slide* + *surrogate variable *(1/1 surrogate variables detected)
	FTLD-Tau (sporadic PSP)			
	Controls (*N* = 70)	76.17 ± 7.93	45M/25F	

FTLD, Frontotemporal lobar degeneration; FTLD-TDP, FTLD with 43 kDa transactive response DNA-binding protein (TDP-43) positive inclusions; FTLD-Tau, FTLD with tau-positive inclusions; PSP, progressive supranuclear palsy; SD, Standard deviation; F, Females; M, Males; Double^−^ proportions, NeuN^−^/SOX10^−^ proportions

### Measures of brain atrophy, neuronal cell loss and pathology-related traits

For FTLD1 and a proportion of FTLD2, formalin-fixed paraffin-embedded (FFPE) sections were also available for more detailed neuropathological evaluations, including sections stained for standard haematoxylin and eosin (H&E). These FFPE sections were from the opposite brain hemisphere with respect to the frozen tissue used for the DNA methylation profiling.

For FTLD1 and FTLD2, microscopic atrophy was assessed on H&E stained slides, by examining the cortical thickness and neuronal loss in the frontal and temporal cortices. A four point grading system was used in comparison to a neurological normal control with no underlying neurodegenerative changes: 0—the cortical thickness was within normal limits and no neuronal loss was observed; 1—reduction in cortical thickness but the number of neurons was comparable to normal levels; 2—reduction in cortical thickness and reduction in the numbers of neurons; 3—severe reduction in cortical thickness and no neurons observed. For each region, the microscopic atrophy was scored semi-quantitatively by an experienced observer blinded to clinical, histopathological and genetic status, at an objective magnification of × 20. Macroscopic atrophy was also determined for FTLD1 based on observations of gyri and sulci from the coronal slices observed during brain cutting procedures. Levels of atrophy were graded, as previously described [70], into four stages: none, mild, moderate, and severe. These neuropathological scores of the frontal and temporal regions were used in the module-trait correlations with the DNA co-methylation network modules.

### DNA methylation profiling and data quality control

For FTLD1, genomic DNA was extracted from carefully dissected flash frozen frontal cortex grey matter tissue using standard protocols. Bisulfite conversion was performed with the EZ DNA Methylation Kit (Zymo Research) using 500 ng of genomic DNA. For FTLD2 and FTLD3, frontal lobe DNA extractions and bisulfite conversions were performed previously as described by Menden et al. [54] and Weber et al. [84]. Genome-wide methylation profiles were generated using the Infinium HumanMethylationEPIC BeadChip (Illumina) for FTLD1 and FTLD2, or the Infinium HumanMethylation450 BeadChip (Illumina) for FTLD3, as per the manufacturer’s instructions.

Beta-values ranging from 0 to 1 (approximately 0% to 100% methylation, respectively), were used to estimate the methylation levels of each CpG site using the ratio of intensities between methylated and unmethylated alleles. Data analysis was conducted using several R Bioconductor packages as previously described [11]. All three cohorts were subjected to harmonised quality control checks and pre-processing. Briefly, raw data (idat files) were imported and subjected to rigorous pre-processing and thorough quality control checks using minfi [4], wateRmelon [68], and ChAMP packages [77]. The following criteria were used to exclude probes that did not pass quality control checks from further analysis: (1) poor quality, (2) cross reactive, (3) included common genetic variants, and (4) mapped to *X* or *Y* chromosome. In addition, samples were dropped during quality control if: (1) they presented with a high failure rate (≥ 2% of probes), (2) the predicted sex did not match the phenotypic sex, and (3) they clustered inappropriately on multidimensional scaling analysis. Beta-values were normalised with ChAMP using the Beta-Mixture Quantile (BMIQ) normalisation method. *M*-values, computed as the logit transformation of beta-values, were used for all statistical analysis, as recommended by Du et al. [23], owing to their reduced heteroscedasticity (as opposed to beta-values) and improved statistical validity for differential methylation analysis.

As significant batch effects were detected during quality control checks, and different FTLD subgroups/subtypes were studied in FTLD1-3, the three cohorts were analysed separately first and then meta-analysed. Similarly, co-methylation network analyses were conducted on each cohort separately, and module preservations were then cross-checked with data from the other cohorts (as described in more detail below).

### Cell-type deconvolution based on DNA methylation data

As DNA methylation patterns are often cell-type specific, changes in different brain cell-type proportions constitute an important confounding factor for DNA methylation studies performed on ‘bulk’ brain tissue. We used a novel cell-type deconvolution reference panel recently described by Shireby et al. [73] which brings more granularity and expands previous methods that account only for neuronal (NeuN+) versus all other cell types (NeuN−). This new method uses novel DNA methylation data obtained from fluorescence activated sorted nuclei from cortical brain tissue to estimate the relative proportions of neurons (NeuN+), oligodendrocytes (SOX10+) and other glial brain cell types (Double−[NeuN−/SOX10−]). Cell-type proportions in bulk brain tissue were thus estimated using the CETYGO (CEll TYpe deconvolution GOodness) package (https://github.com/ds420/CETYGO), and the sorted cell-type reference datasets as described by Shireby et al. [73]. Pairwise comparisons between FTLD cases and controls were conducted using Wilcoxon rank sum test with Benjamini–Hochberg correction for multiple testing, and adjusted *p* < 0.05 was considered significant.

### Differential methylation analysis and EWAS meta-analysis

We applied linear regression models (Table 1) using the *M*-values as the input to identify associations between DNA methylation variation at specific CpG sites and FTLD using the limma package [65]. For FTLD1, we have accounted for possible confounding factors, such as age and sex as well as factors detected in principal components 1 and 2 as seen in Singular Value Decomposition (SVD) plots (ChAMP package), which included cell proportions (SOX10 + and Double−) and sample position in the array. Using this regression model, no surrogate variables were detected with the num.sv function of the SVA package [43], meaning there were no remaining unknown, unmodelled, or latent sources of noise [65]. The same process was applied to FTLD2 and FTLD3. The model for FTLD2 was further adjusted for slide, whereas for FTLD3, the model was further adjusted for slide and one surrogate variable (Table 1). False discovery rate (FDR) adjusted *p*-values < 0.05 were considered genome-wide significant.

We used the estimated coefficients and SEs obtained from the regression models, described above for the three FTLD cohorts, to undertake an inverse variance meta-analysis using the metagen function from the meta R package [8]. Only methylation probes present in all datasets (*N* = 363,781) were considered for this analysis. When reporting differentially methylated sites, a conservative Bonferroni significance was defined as *p* < 1.374 × 10^−7^ (*p* < 0.05/363,781) to account for multiple testing. We report random-effects meta-analysis results as the three cohorts included different FTLD subgroups/subtypes according to the neuropathological classification possibly leading to high heterogeneity in the meta-analysis. We also used a less stringent FDR adjusted *p* < 0.10 to report top meta-analysis loci, all of which were then investigated in the co-methylation networks.

### Co-methylation network analysis

To identify clusters of highly correlated CpGs (co-methylation modules) in an unsupervised manner, i.e. agnostic of gene ontology, we used a systems biology approach based on weighted gene correlation network analysis (WGCNA) [39]. For this analysis, we focussed on CpGs present in all three FTLD datasets, non-intergenic CpGs (i.e. CpGs annotated to genes), and selected the top 20% with the highest variance across individuals in each cohort regardless of their disease status (i.e. most variable 56,001 CpG sites per cohort). After outlier exclusion, a total of 23, 42 and 157 samples remained in the FTLD1, FTLD2 and FTLD3 cohorts, respectively. For each network, we used as input the *M*-values adjusted for the covariates included in the models described above (Table 1) and constructed signed networks. Modules were calculated using the WGCNA blockwiseModules function, with a minimum module size of 200 and a soft-thresholding power of 16, 10 and 12 for the FTLD1, FTLD2 and FTLD3 networks, respectively. Module membership (MM) was then reassigned for each network using the applyKMeans function of the CoExpNets package [13]. Highly connected CpGs within a module (hub CpGs) present with high *M*-values to the respective module. In “Results”, we refer to hub CpGs as those with the highest MM within a given module.

Using a principal component analysis on the CpG methylation values within each module, the CpGs inside each module were represented by a weighted average, the module eigengene (ME). The MEs were then correlated with the FTLD status, FTLD subtypes, and other sample traits, including disease onset and duration, measures of macroscopic atrophy and neuronal loss scores, and other pathology-related traits, as available for each cohort.

To gain insights into the biology underlying the FTLD-related modules, we carried out functional enrichment for CpGs mapping to genes using the default parameters of clusterProfiler [86]. We also carried out cell-type enrichment analysis on the FTLD-related modules using the package EWCE [74] and associated single-cell transcriptomic data [89].

### DNA methylation cross-network module preservation analysis

As a method for differential network analysis, i.e. to identify which co-methylation modules in each of the three generated FTLD networks were preserved (i.e. shared) or perturbed (i.e. unique) in the other two datasets, we employed module preservation analysis, as described by Langfelder et al. [40]. For each network (taken as the “reference dataset”), module preservation in the other two datasets (the “test data”) was calculated using the modulePreservation function implemented in WGCNA. In all instances, the “test data” contained methylation values (adjusted *M*-values) for the 56,001 CpG sites used to construct the “reference dataset” network. A total of 200 permutations for each preservation analysis was used. As a measurement of module preservation, we used the *Z*-summary statistic (a composite measure to summarise multiple preservation statistics). A *Z*-summary greater than 10 indicates a strong preservation of this module in the “test data”, a *Z*-summary of between 2 and 10 indicates moderate preservation, and a *Z*-summary less than 2 indicates no preservation.

### Comparisons of DNA methylation hits with FTLD frontal/temporal cortex gene expression data

To examine the gene expression patterns of the EWAS meta-analysis gene hits, we used previously published transcriptomics data from bulk frontal cortex tissue of FTLD-TDP cases and controls [31] as well as bulk temporal cortex tissue of FTLD-tau cases (PSP) and controls [83]. It is of note that a subset of individuals from the FTLD1 DNA methylation cohort [FTLD-TDP (*N* = 14) and controls (*N* = 5)] overlaps with those with transcriptomics data from Hasan et al. [31] [FTLD-TDP (*N* = 80) and controls (*N* = 48)]. To further infer the expression patterns of selected DNA methylation top genes in specific brain cell types, we also correlated gene expression levels (adjusted for age, sex, and RNA integrity number) with cellular proportions using data from Hasan et al. [31], with the cellular proportions having been estimated using the method described by Mathys et al. [51].

### Comparisons of DNA methylation hits with FTLD-TDP frontal cortex proteomics data

To examine the gene expression patterns of the EWAS meta-analysis gene hits at the protein level, we used proteomics data from FTLD-TDP and controls. Briefly, frontal cortex homogenate of frozen post-mortem human brain tissue was prepared from control (*N* = 6), FTLD-TDP type A with *C9orf72* repeat expansion (*N* = 6), and FTLD-TDP type C (*N* = 6) cases, as previously described [58]. Proteins in both the soluble supernatant and the insoluble pellet fraction were analysed, and samples were pooled per disease group (three cases per pooled sample) to enable deeper coverage of the proteome with higher fractionation. Proteins were quantitated using 2D-LCMS and UDMSe label-free proteomics and SYNAPT G2-Si High Definition mass spectrometer operating in ion mobility mode. Data were processed using Progenesis software, as previously described [79]. A total of 6114 proteins were detected in the supernatant, and 5108 in the pellet, with an overlap in some proteins that were found both in the supernatant and pellet. Fold-changes between FTLD-TDP subtypes compared to controls were calculated. Of the Bonferroni significant EWAS meta-analysis hits, only the OTUD4 protein was detected (both in the supernatant and in the pellet).

### Comparisons of DNA methylation hits with additional datasets

We further investigated the normal expression patterns of the meta-analysis gene hits both in the human and mouse brains using single-nuclei RNAseq data from the Allen Brain Map (https://celltypes.brain-map.org/) [7], and data from the Allen Mouse Brain Atlas (http://mouse.brain-map.org) [44]. Given the *OTUD4*-related findings, we investigated the list of cortical tissue OTUD4 protein interactors made available by Das et al. [20]. The RNA granule database (http://rnagranuledb.lunenfeld.ca/) collates curated literature evidence that support gene or protein association with the stress granules (SGs) and P-bodies (PBs). We used a list of tier 1 genes from the RNA granule database version 2.0 for comparisons with the lists of genes composing the three *OTUD4* FTLD-associated co-methylation modules.

### OTUD4 immunohistochemical staining

To investigate tissue expression patterns of OTUD4 protein across the human cortex, FFPE frontal cortex tissue from 7 FTLD cases (4 FTLD-TDP type A and 3 FTLD-TDP type C) and 3 controls (overlapping with FTLD1) were utilised. Briefly, 8-mm-thick sections cut from the FFPE blocks were immunostained using a standard avidin–biotin-peroxidase complex method with di-aminobenzidine as the chromogen [41]. The rabbit anti-OTUD4 antibody (Atlas Antibodies HPA036623, 1:200) was used, along with heat antigen retrieval pre-treatment prior to application of the primary antibody. The samples were mounted and examined using a light microscope.

## Results

### Cell-type deconvolution based on DNA methylation data highlights important cellular composition differences in FTLD

**Fig. 2:**
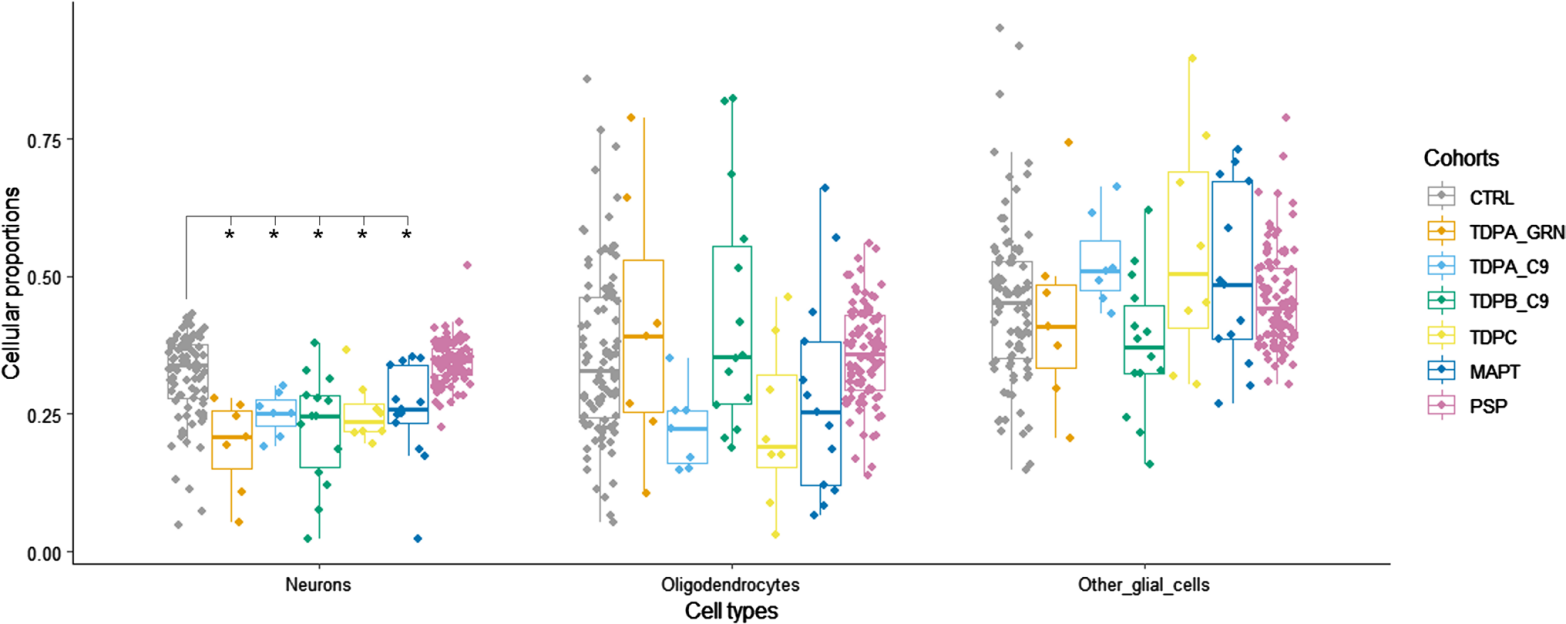
Brain cell-type proportion estimates derived from bulk DNA methylation data in frontal lobe of frontotemporal lobar degeneration (FTLD) and controls. *Indicates significant differences for each cell-type between FTLD subtypes and the corresponding controls; pairwise comparisons were performed using the Wilcoxon rank sum test, and adjusted *p*-values < 0.05 were considered significant. CTRL, controls; TDPA_GRN, FTLD with TDP-43 positive inclusions (FTLD-TDP) subtype A, carriers of *GRN* mutations; TDPA_C9, FTLD-TDP subtype A, carriers of *C9orf72* repeat expansion; TDPB_C9, FTLD-TDP subtype B, carriers of *C9orf72* repeat expansion; TDPC, FTLD-TDP subtype C, sporadic; MAPT, FTLD with tau-positive inclusions (FTLD-Tau), carriers of *MAPT* mutations; PSP, FTLD-Tau, sporadic progressive supranuclear palsy; Neurons, NeuN + ; Oligodendrocytes, SOX10 + ; other glial cells, NeuN−/SOX10−

To estimate brain cell-type proportions in our bulk frontal cortex DNA methylation datasets, we used a refined cell-type deconvolution algorithm based on reference DNA methylation profiles from purified nuclei from neurons (NeuN+), oligodendrocytes (SOX10+) and other brain cell types (NeuN−/SOX10−) [73]. This new model controls better for cellular heterogeneity in bulk cortex tissue compared to previous models, which account only for neuronal (NeuN+) versus all glial cells (NeuN−). Within each sample group, we observed extensive variability in cell-type proportions across cell types (Fig. 2). When comparing disease cases with controls, no overall differences were detected in the proportions of oligodendrocytes (SOX10+) and other glial cells (NeuN−/SOX10−) after accounting for multiple testing corrections. However, with the exception of the PSP cases (FTLD3), all FTLD subgroups/subtypes showed a significant decrease in neuronal proportions compared to controls (Wilcoxon rank sum test, adjusted *p* < 0.05), as expected in neurodegenerative diseases. These findings highlight the importance of adjusting for cell-type proportions in bulk tissue EWAS studies. Accounting for this allowed us to identify DNA methylation changes that are relevant to the disease rather than merely reflecting changes in cell-type composition, which could be related partly to the disease pathogenesis itself and partly due to technical issues (e.g. a result of capturing different proportions of grey and white matter during tissue dissection).

### Frontal cortex case–control EWAS meta-analysis identifies shared differentially methylated CpG sites across FTLD pathological subgroups and subtypes

First, we investigated DNA methylation variation in specific loci across the genome as covered by the 450K/EPIC arrays, using linear regressions models to perform cohort-specific case–control EWAS. For FTLD1 and FTLD2, which comprise heterogeneous cases with sporadic and genetic forms of FTLD-TDP and FTLD-tau pathology, no genome-wide significant CpGs were identified. For FTLD3, which only includes cases with FTLD-tau pathology (sporadic PSP), 234 differentially methylated positions were identified (Supplementary Table S1, Online Resource). The top differentially methylated CpG in the FTLD3 cohort was cg09202319, which was hypomethylated in FTLD-tau (PSP) compared to controls (adjusted *p* = 6.54 × 10^–8^). This CpG mapped to a CpG island in the promoter region of *PFDN6* (Prefoldin Subunit 6), which is involved in promoting the assembly of cytoskeletal proteins [45]. Supplementary Fig. S1 (Online Resource) shows the quantile–quantile (Q–Q) plots for each of the single cohort-specific EWAS.

**Fig. 3:**
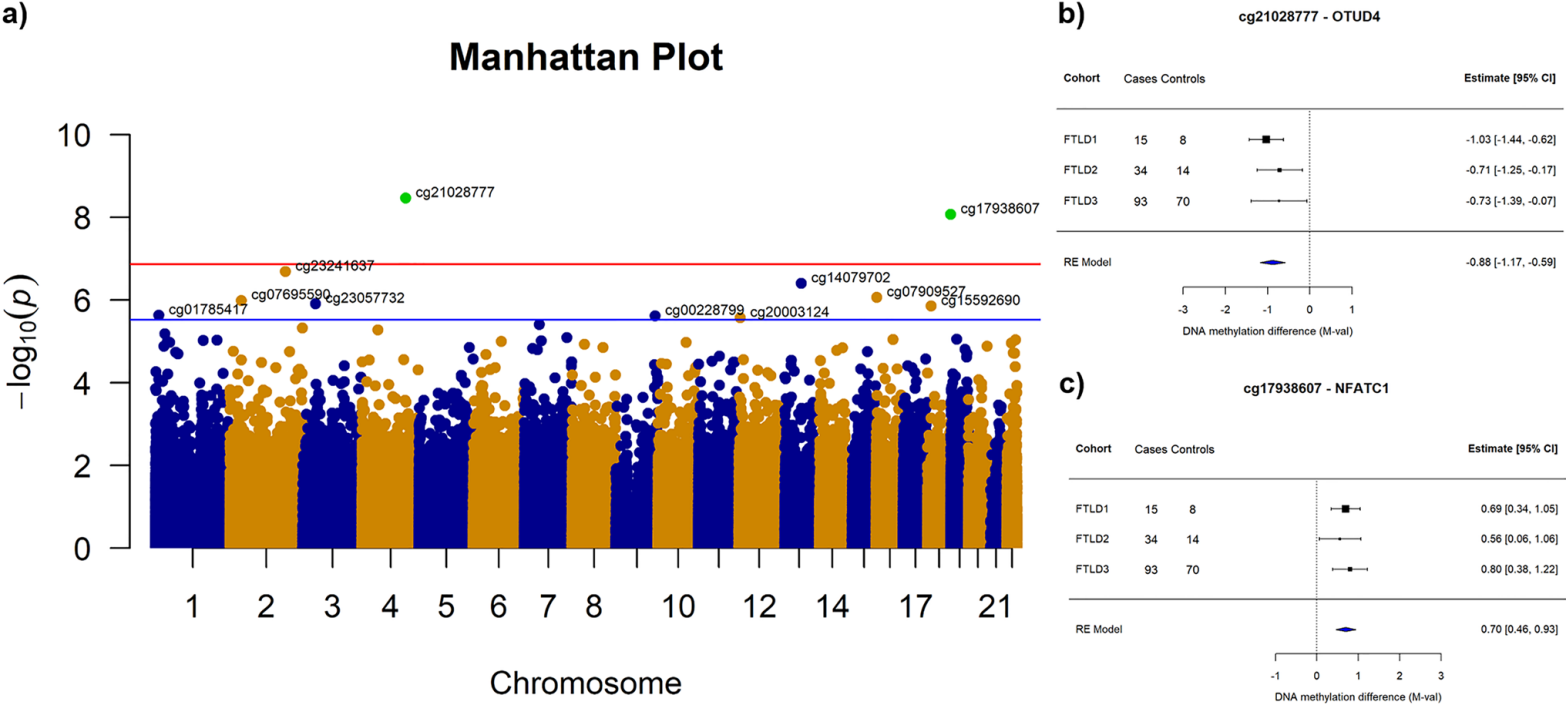
Differentially methylated positions identified in a case–control FTLD cross-cohort EWAS meta-analysis. **a** Manhattan plot showing associations between single DNA methylation sites (CpGs) and FTLD from the EWAS meta-analysis random-effect results (total *N* = 234). CpGs are plotted on the *x*-axis according to their positions on each chromosome against association with FTLD on the *y*-axis (− log 10 *p*-value). The top red line indicates the conservative Bonferroni significance threshold (α) of *p* = 1.37 × 10^−7^. Green points indicate CpGs passing the Bonferroni threshold. The blue line indicates a less stringent threshold of *p* = 2.70 × 10^−6^ (FDR *p* = 0.10). **b** Forest plot depicting the CpG in *OTUD4*, which is significantly hypomethylated in FTLD compared to controls in the cross-cohort meta-analysis (FTLD1 *N* = 23, FTLD2 N = 48, and FTLD3 *N* = 163). **c)** Forest plot depicting the CpG in *NFATC1*, which is significantly hypermethylated in FTLD compared to controls in the cross-cohort meta-analysis (FTLD1 *N* = 23, FTLD2 *N* = 48, and FTLD3 *N* = 163)

Second, we meta-analysed the single cohort EWAS results, enabling an analysis of FTLD-associated differential cortical DNA methylation using tissue from 234 individuals (142 FTLD cases and 92 controls). After a conservative Bonferroni adjustment for multiple testing (*p* < 1.37 × 10^− 7^), the meta-analysis identified two differentially methylated CpGs in FTLD compared to controls, regardless of the pathological subgroup (FTLD-TDP or FTLD-tau), and corresponding subtypes (Fig. 3; Supplementary Fig. S2, Supplementary Table S2, Online Resource). The top CpG was annotated to a shore in the 5’UTR of *OTUD4* and was hypomethylated in FTLD compared to controls, whereas the other was annotated to a CpG island in the body of *NFATC1* and hypermethylated in FTLD compared to controls (Fig. 3). The direction of the effect was consistent across the three FTLD cohorts for these two hits, as well as for nine additional top meta-analysis loci obtained when considering a less stringent FDR *p* < 0.10 multiple testing correction (Fig. 3; Supplementary Table S2, Online Resource). Of note, none of these meta-analysis top differentially methylated sites showed epigenome-wide significant changes in FTLD3 alone (Supplementary Table S1, Online Resource) or in previous Alzheimer’s disease EWAS meta-analyses (Supplementary Table S2, Online Resource).

### Frontal cortex FTLD EWAS meta-analysis hits are consistent with downstream changes in mRNA and protein expression patterns

**Fig. 4:**
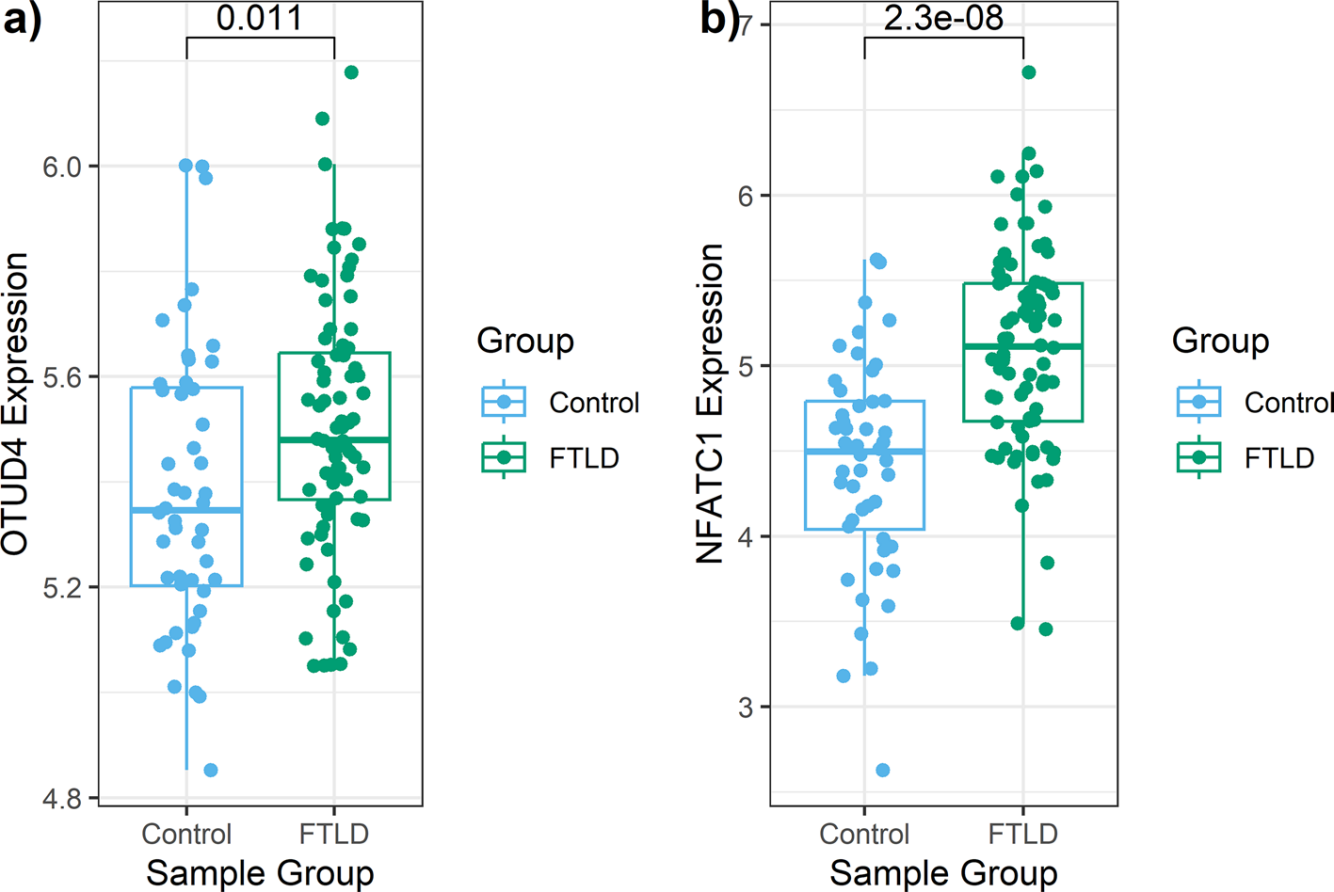
Boxplots showing gene expression levels in the frontal cortex for the two EWAS meta-analysis hits in FTLD-TDP and controls. RNA sequencing data from Hasan et al. [31] adjusted for age, sex, and RNA integrity number was used. Log2-transformed gene expression data is shown in the *y*-axis, and non-paired *t*-test *p*-value for the comparison between FTLD-TDP (*N* = 80) and controls (*N* = 48) is denoted at the top

To explore possible downstream consequences of DNA methylation variation on gene expression in FTLD, we investigated available FTLD-TDP and FTLD-tau transcriptomic data [31, 83], as well as FTLD-TDP proteomics data. From the EWAS meta-analysis hits passing Bonferroni correction, consistent results were observed in both FTLD-TDP (frontal cortex) and FTLD-tau (temporal cortex) for *OTUD4*, which showed higher mRNA expression levels in FTLD cases compared to controls (Fig. 4). When considering FTLD-TDP gene expression data from Hasan et al. [31] only for a subset of cases and controls with overlapping FTLD1 DNA methylation data (*N* = 19), the *OTUD4* gene expression patterns were very similar to the whole dataset (*N* = 128). For *NFATC1*, increased expression was observed in FTLD-TDP when compared to controls (Fig. 4). However, this increase in expression was not observed in the FTLD-Tau, as seen in data from Wang et al. [83]. Of the nine additional top meta-analysis loci, *ZNF804A* and *KLF12* showed lower mRNA expression levels and *IMPA2* showed higher mRNA expression levels in FTLD cases compared to controls (*p* < 0.05, Supplementary Fig. S3, Online Resource). DNA methylation levels in upstream regulatory regions are often inversely associated with gene expression levels [61, 80]. Therefore, lower methylation levels in CpGs annotated to 5’UTR in *OTUD4* and to TSS200 in *IMPA2*, and higher expression of these genes in FTLD compared to controls, meets such expectations. On the other hand, DNA methylation levels in gene bodies are usually positively associated with gene expression. Again, results align with this in the case of *NFATC1* (which showed higher methylation and higher expression in FTLD-TDP compared to controls) and *ZNF804A* (which showed lower methylation and lower expression in FTLD).

**Fig. 5:**
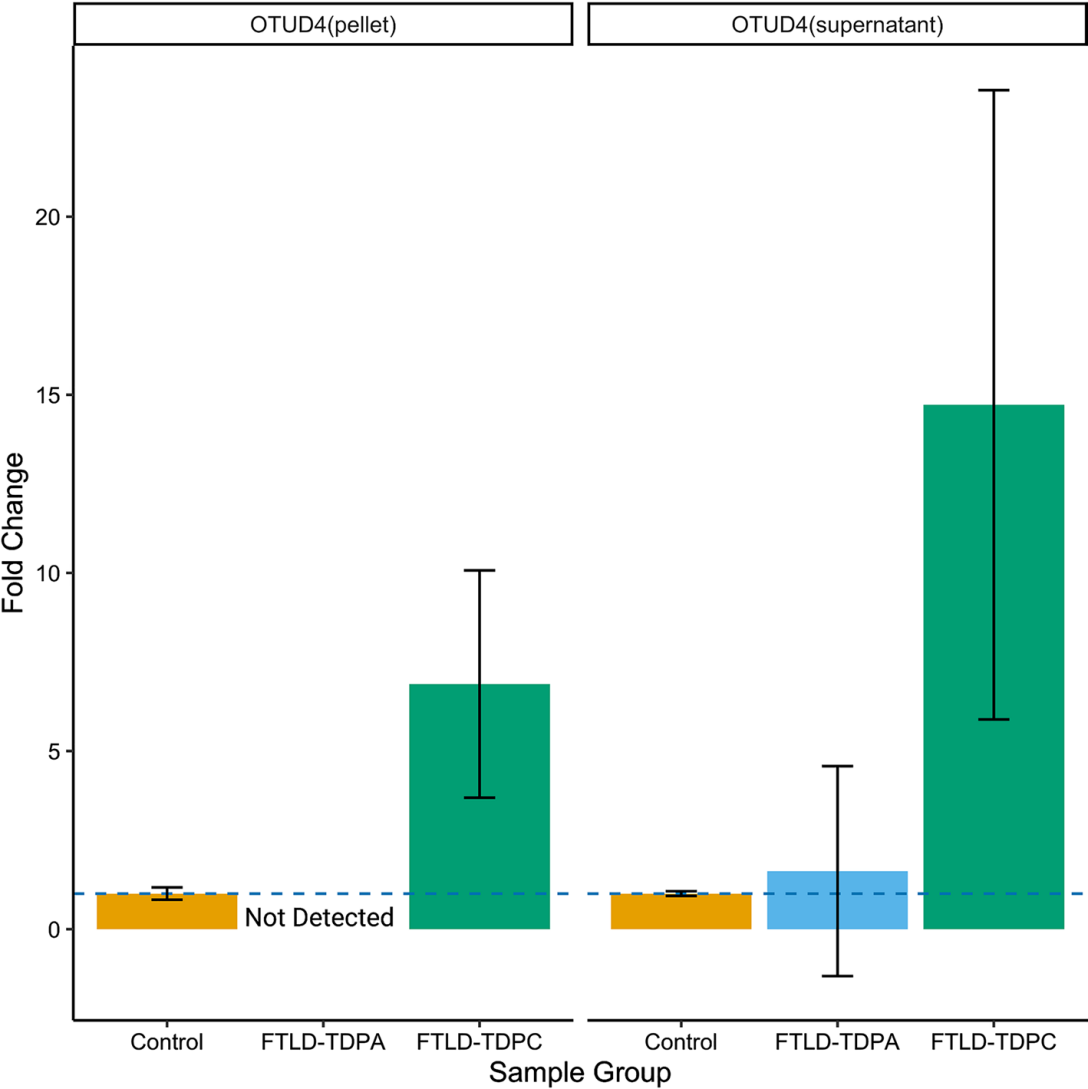
Bar plots of protein quantifications in the frontal cortex for the EWAS meta-analysis hit *OTUD4* in FTLD-TDP subtypes and controls. Out of the two EWAS meta-analysis hits, only the OTUD4 protein was detected in the proteomics data and is presented here. OTUD4 was detected in both fractions (pellet and supernatant). Two pooled samples (2 × 3 samples) per group were analysed. Protein levels were quantitated using mass spectrometry. The average values were obtained for each group, and fold-changes were calculated comparing FTLD-TDP subtypes with controls. Bar plots show mean fold-change and corresponding standard error

**Fig. 6:**
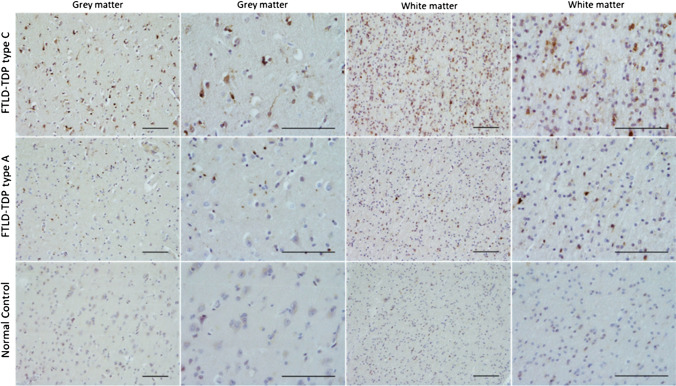
Immunoreactivity of OTUD4 in the frontal cortex of FTLD-TDP (*N* = 4 type A and *N* = 3 type C) and controls (*N* = 3). Immunohistochemical analysis was carried out in FFPE frontal cortex tissue from FTLD-TDP cases and controls overlapping with FTLD1, using a rabbit anti-OTUD4 antibody (Atlas Antibodies HPA036623, 1:200). Scale-bars represent 100 µm

Only one of the two Bonferroni adjusted meta-analysis gene hits were detected in the frontal cortex proteomics data. OTUD4 protein was upregulated in FTLD-TDP in types A and C compared to controls (Fig. 5), with the highest fold-change being observed in type C for the supernatant soluble fraction (fold-change = 14.72). These findings are in line with our observations from the RNAseq data and support consistent dysregulation of the *OTUD4* EWAS meta-analysis hit in FTLD. Therefore, we further investigated the patterns of OTUD4 protein expression in the frontal cortex and performed anti-OTUD4 immunohistochemical analysis (Fig. 6) using FTLD-TDP types A and C cases as well as controls that overlap with those used in the DNA methylation analysis (subset of the FTLD1 cohort). Minimal neuronal cytoplasmic staining was observed in the normal controls. However, in the FTLD-TDP cases, an increase in cytoplasmic staining intensity was observed in both the grey and white matter. In the grey matter, neuronal cytoplasmic staining was seen together with glial nuclear staining. In the white matter, there was an increase in glial staining. These results concur with the results from our proteomics and transcriptomics data.

### DNA co-methylation modules are associated with the FTLD status, FTLD pathological subtypes, and disease-related traits

To provide insight into higher order relationships across DNA methylation sites (CpGs), we used an agnostic systems biology approach based on WGCNA and constructed co-methylation networks. Considering the top 20% most variable CpGs in each of the 3 cohorts (*N* = 56,001 CpGs), we identified clusters of highly correlated CpGs, henceforth called co-methylation modules, each assigned a colour name.

**Fig. 7:**
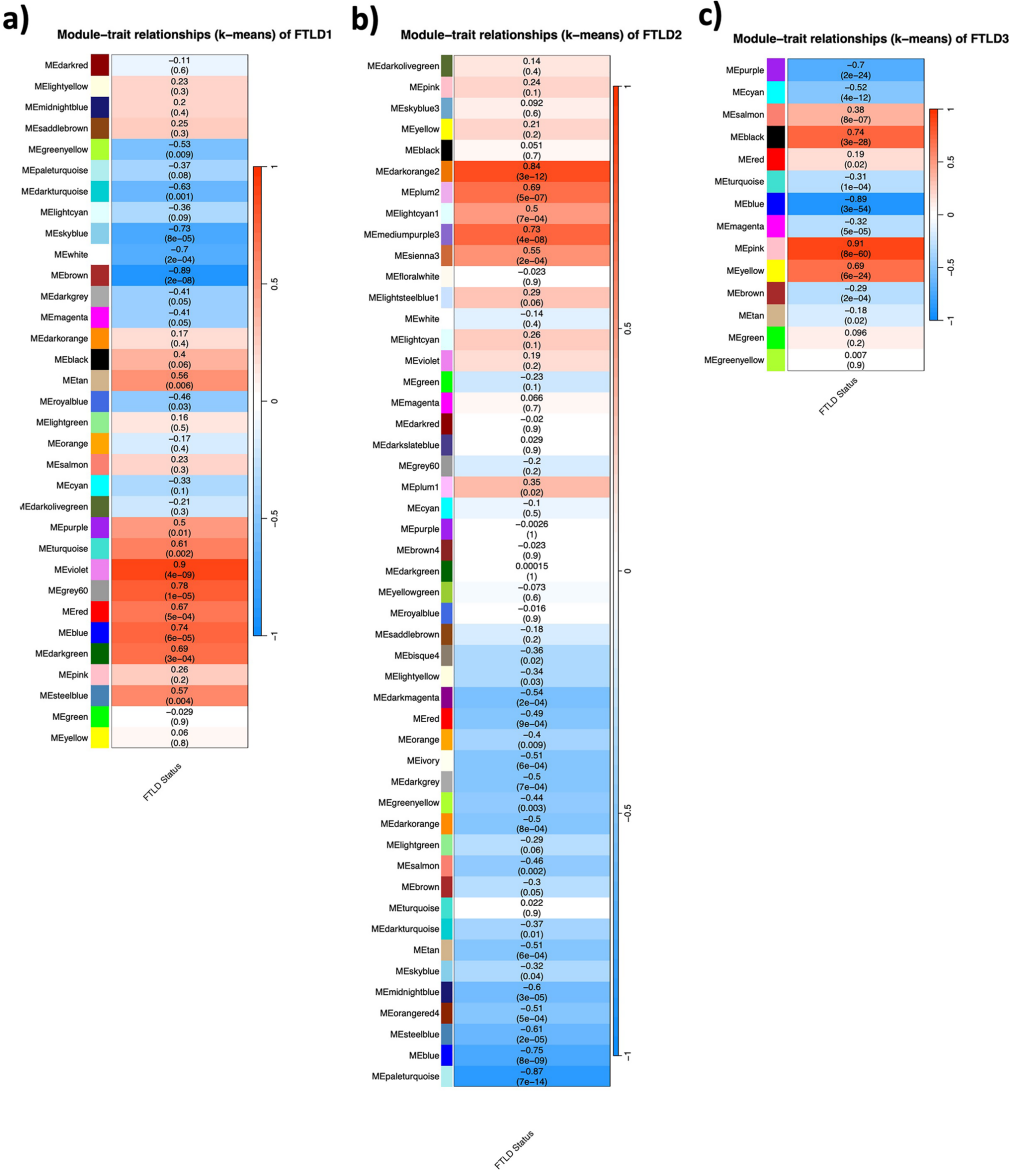
Module-trait correlations for the FTLD co-methylation networks. **a** FTLD1; **b** FTLD2; **c** FTLD3. The rows represent the co-methylation module eigengenes (ME) and their colours, and the column represents the correlation of the methylation levels of CpGs in each module with the disease status. *p*-values are presented within each cell and the colour scale at the right indicates the strength of the correlation (darker cells depict stronger correlations, with blue representing negative and red representing positive correlations)

For the FTLD1, FTLD2 and FTLD3 networks, 9/33 (*p* < 0.002, 0.05/33 modules), 16/49 (*p* < 0.001, 0.05/49 modules) and 10/14 (*p* < 0.004, 0.05/14 modules) co-methylation modules were found to be associated with the disease status (i.e. FTLD or control), respectively (Fig. 7a–c). Our co-methylation network analysis also revealed modules associated with specific pathological subgroup/subtypes in FTLD1 and FTLD2 networks (Supplementary Fig. S4, Online Resource). In a few cases, opposite effect directions were shown in one subgroup/subtype compared to another (e.g. midnightblue and salmon modules in FTLD1 TDPA versus TDPC, Supplementary Fig. S4a; and turquoise module in FTLD2 TDP versus Tau, Supplementary Fig. S4b; Online Resource). More detailed identification of subtype-specific DNA methylation signatures warrants further investigation in the future studies.

We also tested for correlations with additional disease-related traits as available for FTLD1, FTLD2, and FTLD3. We found associations between FTLD-associated co-methylation modules and disease duration as well as with macroscopic and/or microscopic measures of atrophy/neurodegeneration in the frontal and temporal lobes (Supplementary Fig. S4a, b, Online Resource). Two out of the ten modules associated with the disease status in FTLD3 were also associated with tau pathological burden (Braak stage, Supplementary Fig. S4c, Online Resource).

To assess replication of FTLD-associated co-methylation modules across datasets, we then ran preservation analysis for each dataset against each of the networks. We found that most of the FTLD-associated co-methylation modules were indeed moderately to highly preserved (*Z*-summary > 2) in at least one of the other two datasets (Supplementary Fig. S5, Online Resource), further supporting their relevance to FTLD regardless of the pathological subgroup/subtype. Exceptions to this were observed only for the FTLD1 brown, darkturquoise and grey60, and the FTLD2 darkorange2 modules, which seem to be perturbed in the other two datasets.

### Genes that compose FTLD-associated co-methylation modules are involved in transcription regulation, phosphorylation, the ubiquitin system and actin cytoskeleton dynamics

**Fig. 8:**
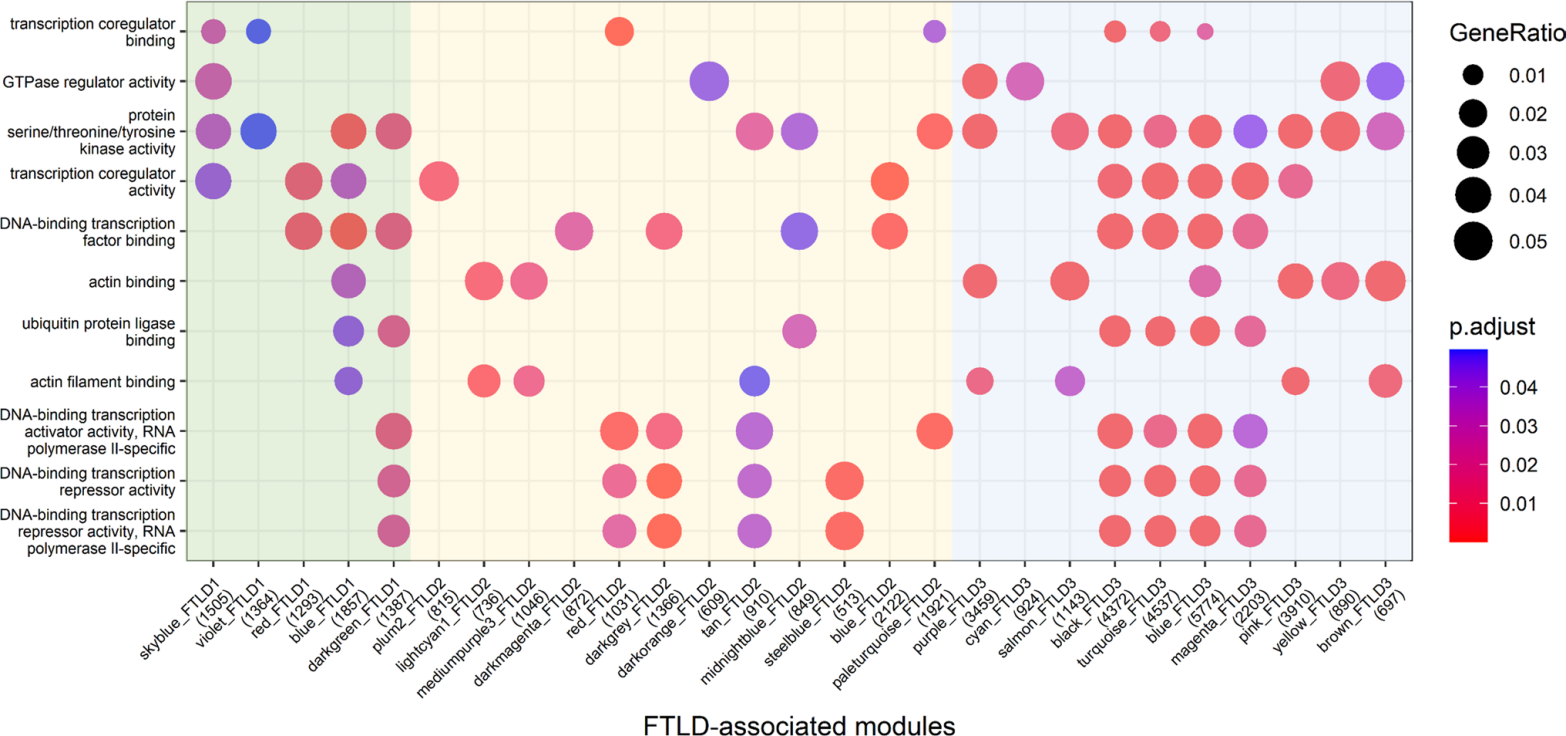
Functional enrichment for the FTLD-associated co-methylation modules across the three networks. *y*-axis shows top enriched gene ontology terms, while *x*-axis depicts FTLD-associated modules in FTLD1 (green), FTLD2 (yellow) and FTLD3 (blue) co-methylation networks. Modules not showing enrichment for shared terms across the networks are not shown

We then performed functional enrichment analysis to investigate which gene ontologies were shared across the three FTLD co-methylation networks. We found significant enrichment of terms related with transcription regulation (e.g. “DNA-binding transcription factor binding”), phosphorylation (“protein serine/threonine/tyrosine kinase activity”), the ubiquitin system (e.g. “ubiquitin protein ligase activity”), and actin cytoskeleton dynamics (e.g. “actin filament binding”). This was observed across the three co-methylation networks and across different modules of each network (Fig. 8). Dysregulation of all these processes had been previously linked to FTLD [69], and our findings now support a role for DNA methylation as a mechanism involved in such dysregulation.

### FTLD-associated modules are enriched for genes relevant for pyramidal neurons and endothelial cells across all three co-methylation networks

**Fig. 9:**
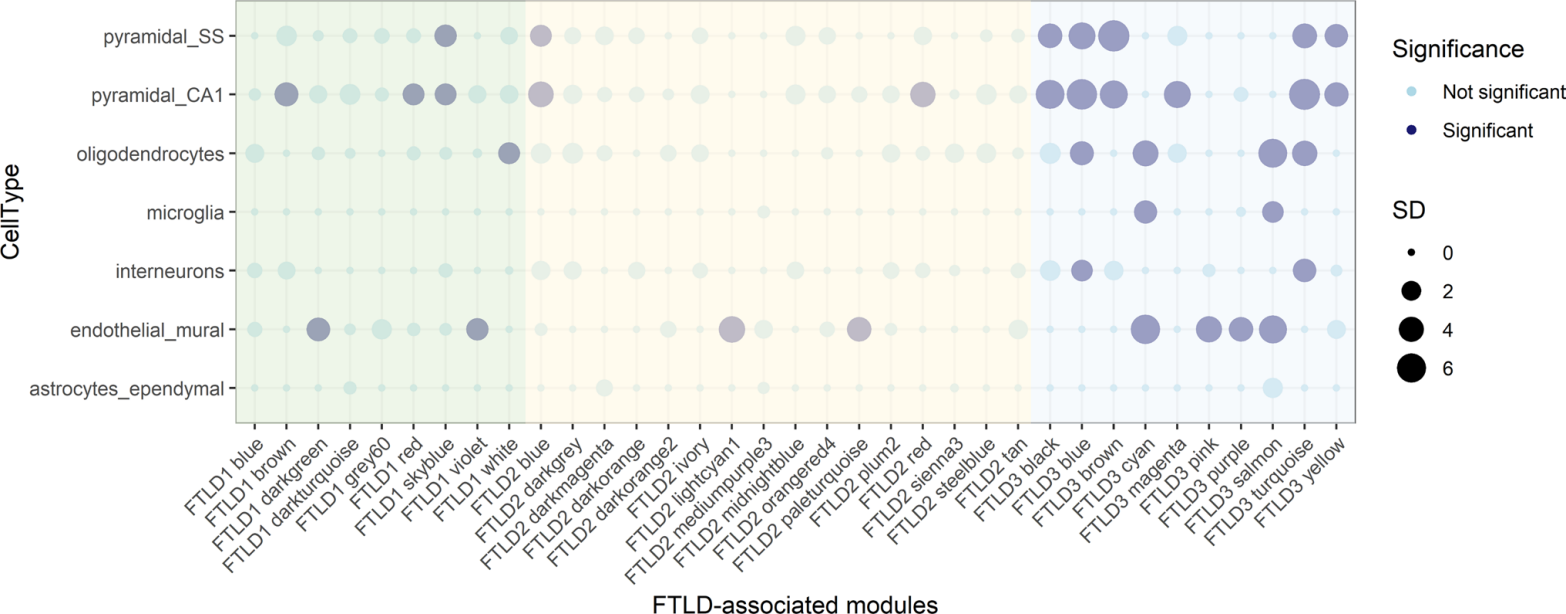
Cell-type enrichment for all FTLD-associated co-methylation modules across the three co-methylation networks. Green denotes FTLD-associated modules in the FTLD1 network; Yellow denotes FTLD-associated modules in the FTLD2 network; Blue denotes FTLD-associated modules in the FTLD3 network. Dark filled circles highlight the cell types found to be significantly enriched with adjusted *p* < 0.05 after Bonferroni correction over all cell types within each module; the size of the circles represents the number of standard deviations (SD) from the mean. Cell-type enrichment analysis on the FTLD-related modules was performed using the package EWCE [74] and associated single-cell transcriptomic data [89]

We also aimed to elucidate whether the genes that compose FTLD-associated co-methylation modules are relevant for specific brain cell types. Across the three networks (FTLD1, FTLD2 and FTLD3), we found significant enrichments for pyramidal neurons and endothelial/mural cells (Fig. 9), suggesting these cell types are consistently affected by the DNA methylation changes in FTLD regardless of the pathological subgroup/subtype. Previous studies with pathological assessment, as well as transcriptomic analysis in FTLD brain tissue, support changes in these cell types in FTLD [25–27, 31, 64]. In addition, in the FTLD1 and the FTLD3 networks, we found signatures with an overrepresentation of oligodendrocyte markers. Of note, FTLD3 is composed of PSP cases, which, unlike the other FTLD groups studied here, is known to present with pathological accumulation of tau in the oligodendrocytes [85]. The FTLD3 network was also enriched for microglia and interneurons.

### *OTUD4* and other top meta-analysis loci are co-methylated in all three networks

We then examined whether the 11 EWAS meta-analysis top loci (FDR *p* < 0.10) were present (Supplementary Table S2, Online Resource) in the co-methylation networks and whether any co-methylation modules were enriched for such loci (Supplementary Table S3, Online Resource). Notably, the top meta-analysis hit in *OTUD4* was present in all three networks (FTLD1—brown, FTLD2—blue, and FTLD3—blue modules), and was always co-methylated with the CpG annotated to *CEBPZ* (Supplementary Table S3, Online Resource). These modules showed a significant enrichment for the top EWAS meta-analysis loci [Fisher’s exact test, FTLD1—brown odds ratio (OR) = 14.9, *p* = 0.003; FTLD2—blue OR = 10.6, *p* = 0.007; FTLD3—blue OR = 8.0, *p* = 0.017). We, therefore, decided to further investigate similarities across these three modules (FTLD1—brown, FTLD2—blue, and FTLD3—blue), which will henceforth be referred to as “*OTUD4*-modules”.

It is of note that only eight CpGs were shared across the three “*OTUD4*-modules”, two of which—cg21028777 in *OTUD4* and cg07695590 in *CEBPZ*—correspond to top EWAS meta-analysis loci (Supplementary Fig. S6, Online Resource), highlighting their importance across the FTLD subgroups/subtypes. All three “*OTUD4*-modules” were inversely related with the disease status, i.e. lower levels of methylation in CpGs composing these modules are associated with increased risk of FTLD (Fig. 7; FTLD1—brown *r* = − 0.89, *p* = 2 × 10^–8^; FTLD2—blue *r* = − 0.75, *p* = 8 × 10^–9^; and FTLD3—blue *r* = − 0.89, *p* = 3 × 10^–54^). FTLD2-blue was also inversely associated with the severity of neuronal loss in the frontal cortex (*r* = − 0.48, *p* = 0.001, Supplementary Fig. S4b, Online Resource). Although not reaching statistical significance after accounting for multiple testing corrections, a similar trend was observed with the severity of neuronal loss in the temporal cortex for FTLD2-blue (*r* = − 0.46, Supplementary Fig. S4b, Online Resource) as well as for FTLD1-brown in both frontal and temporal cortices (*r* = − 0.29 n.s., and *r* = − 0.63 *p* = 0.001, respectively, Supplementary Fig. S4a, Online Resource). These findings further support the relevance of these signatures enriched for top EWAS meta-analysis loci, including CpGs in *OTUD4* and *CEBPZ*, in disease progression/severity.

Previous studies have shown that OTUD4 [20], tau [5], TDP-43, and a growing number of additional FTLD-related RNA-binding proteins [10] play an important role in the biology of stress granules. We, therefore, investigated whether stress granules proteins and OTUD4 protein interactors were present in the “*OTUD4*-modules”. Indeed, many genes encoding for such proteins were represented in these modules, including several genes associated with genetic FTLD risk such as *MAPT* (encoding for tau), present across the three “*OTUD4*-modules”, and *FUS*, present in FTLD3-blue (Supplementary Tables S4 and S5, Online Resource). The same was true for many hnRNPs, such as *HNRNPA1*, *HNRNPC*, and *HNRNPUL1*, which are present in the “*OTUD4*-modules” and are OTUD4 protein interactors (Supplementary Tables S4 and S5, Online Resource). These hnRNPs are also known targets of the transcription factor *CEBPZ* (as described by Ma’ayan et al. [66]), which is also a top EWAS meta-analysis loci and is co-methylated with *OTUD4* across the networks.

We also identified the hub genes in the three “*OTUD4*-modules” (i.e. the most interconnected genes within the module). These were *ADCY1*, *TLE6* and *GDAP1* for FTLD1-brown, FTLD2-blue and FTLD3-blue, respectively (Supplementary Table S4, Online Resource). Of note and highly relevant for FTLD, *ADCY1* has been found to be implicated in learning, memory, and behaviour [72]. The importance of *TLE6* to brain-related disease is supported through its association with bipolar disorder [22], and mutations in *GDAP1* cause inherited peripheral neuropathies [62].

### “*OTUD4*-modules” implicate glutamatergic synapse and pyramidal neurons

More detailed gene ontology enrichment of “*OTUD4*-modules” once again highlighted transcriptional regulation and the ubiquitin system, as well as nuclear speck, synapse (particularly glutamatergic synapse), and axon development (Supplementary Fig. S7, Online Resource). All three “*OTUD4*-modules” showed an enrichment for pyramidal neurons and the FTLD3-blue module additionally showed an enrichment for oligodendrocytes (Fig. 9). Further supporting the importance of *OTUD4* and *CEBPZ* in glutamatergic cells, in the normal brain (human and mouse) these genes show the highest expression in glutamatergic neurons and/or cortical and hippocampal pyramidal and granule cell layers (Supplementary Figs. S8–S9, Online Resource).

Using gene expression data and derived cellular proportions from Hasan et al. [31], we observed a positive relationship between both *OTUD4* and *CEBPZ* expression and proportions of excitatory neurons in controls and FTLD-TDP type A (Supplementary Fig. S10, Online Resource). This finding further supports the relevance of *OTUD4* and *CEBPZ* in excitatory glutamatergic neurons. However, that relationship is perturbed in FTLD-TDP type C (Supplementary Fig. S10, Online Resource), which could suggest higher expression of these genes by fewer surviving excitatory neurons and/or higher expression by other cell type(s).

## Discussion

We have conducted, to our knowledge, the first FTLD EWAS meta-analysis utilising three independent cohorts and incorporating results from 234 brain donors (142 FTLD cases and 92 controls). We identified two differentially methylated CpGs shared across a range of FTLD subgroups (FTLD-TDP and FTLD-tau) and corresponding subtypes, which map to *OTUD4* and *NFATC1*. Systems biology approaches such as co-methylation network analysis are powerful methodologies for identifying pathways and networks which may be more relevant to disease pathophysiology than individual genes. We, therefore, performed a co-methylation network analysis in each of the independent cohorts and identified modules associated with the FTLD disease status and FTLD-related traits. Interestingly, *CEBPZ* always clustered with *OTUD4*, and the “*OTUD4*-modules” were enriched for meta-analysis top loci in each of the three independent cohorts. Using functional and cell-type enrichment analysis of modules of interest, we identified several biological processes with relevance to FTLD pathology, including the ubiquitin system, RNA granule formation and glutamatergic synaptic signalling, which we discuss below. It is of note that none of the loci identified in our meta-analysis match with neuropathology-associated loci identified in large AD studies [73, 75, 90], therefore, supporting the hypothesis that molecular changes in these loci reflect shared disease biology aspects of FTLD subgroups/subtypes rather than a mere downstream consequence of neurodegeneration.

The *OTUD4* gene encodes the protein OTUD domain-containing protein 4, a de-ubiquitinating enzyme [55]. Mutations in this gene are associated with Gordon Holmes syndrome, which is characterised by ataxia and hypogonadotropism [49]. Interestingly, a combination of mutations in *OTUD4* along with mutations in *RNF216*, which codes for a ubiquitin ligase, was also found to result in dementia [49]. The protein is known to have roles in modulating inflammatory signalling [92] and in the alkylation damage response [91], and has more recently been demonstrated to interact with RNA-binding proteins (RBPs), including TDP-43 (which aggregates in FTLD-TDP), which are important in the functioning of neuronal RNA granules and stress granules [18]. RNA granules are structures which facilitate the translocation and storage of mRNAs [38], whilst stress granules are formed when cellular stressors such as oxidative stress are present, possibly as a mechanism to reversibly block translation initiation until the stress has been removed [16, 36]. Notably, similarly to TDP-43 [6], OTUD4 was shown to be important in the correct formation of stress granules [20]. Indeed, there is much evidence as to the importance of the ubiquitin system in the functioning of stress granules [37, 56, 78]. The hypomethylation of the 5’UTR region of *OTUD4* (cg21028777), which was observed as the top hit from the FTLD EWAS meta-analysis, and the inclusion of this CpG in three modules where decreased methylation was associated with increased risk of FTLD indicates that decreased methylation of this gene might be involved in the pathogenesis of FTLD. Further supporting these findings, the *OTUD4* gene and protein expression levels are dysregulated in FTLD [31, 83].

Also supporting the importance of the role of ubiquitination and granule formation are the results from the functional enrichment analysis of the three network modules containing *OTUD4*, which revealed an overrepresentation of terms relating to the ubiquitin system. All three “*OTUD4*-modules” contained terms such as “ubiquitin protein ligase activity”, the FTLD2-blue module also showed enrichment of the GO term “ribonucleoprotein granule”, indicating that other genes in this module might also have processes relevant to granule formation, as with the meta-analysis hit *OTUD4.* Ubiquitin signalling is well described as a process implicated in neurodegenerative disease pathology, and several genes involved in ubiquitin and ubiquitin binding processes are known to be mutated/contain risk alleles in multiple neurodegenerative diseases, including FTD [69].

Ontology terms enriched in our functional analysis of FTLD-associated modules also include many relating to regulation of transcription such as “DNA-binding transcription factor binding” and “transcription coregulator activity”. Another meta-analysis top loci was annotated to the *CEBPZ* gene, which encodes the CCAAT Enhancer Binding Protein Zeta, a transcription factor implicated in cellular response to environmental stimuli through transcriptional processes that regulate heat-shock factors, including HSP70 [48]. HSP70 is a heat-shock protein involved in several protein folding processes, including the refolding of aggregated proteins [33, 47, 63]. Furthermore, HSP70 has been shown to have a role in the prevention of build-up of misfolded proteins in stress granules [50]. Interestingly, a CpG in *PFDN6* was the top-most differentially methylated CpG in the FTLD3 (FTLD-tau) EWAS. This gene encodes for the subunit 6 of prefoldin, which is a co-chaperone of HSP70, regulates the correct folding of proteins and is involved in the proper assembly of cytoskeletal proteins [45]. Prefoldin proteins themselves have also been associated with neurodegenerative disease pathology [45, 76].

Our functional enrichment analysis of the “*OTUD4*-modules”, FTLD1-brown, FTLD2-blue and FTLD3-blue, showed that these modules were enriched for gene ontology terms (for cellular component) relating to synapses, including “synaptic membrane”, “asymmetric synapse”, “postsynaptic density”, and “glutamatergic synapse”. Cell-type enrichment analysis revealed that these three modules were also significantly enriched for markers of pyramidal/glutamatergic cells. These findings were further substantiated with expression patterns of *OTUD4* and *CEBPZ* in single-nuclei and mouse expression data. Glutamate, which is the most abundant excitatory neurotransmitter in the human brain [93], is typically associated with memory, learning and other higher cognitive functions [12], and has also been implicated in neurodegeneration [59]. The contribution of neurotransmitter deficits, and specifically, changes in glutamate and glutamate signalling have been described in FTD [2, 14, 30, 34, 57]. DNA methylation has previously been suggested to be an important regulator of glutamatergic synaptic scaling (also known as homeostatic synaptic plasticity), with demethylation found to be associated with increased glutamatergic synapse strength in cultured neurons [53], we here find evidence supporting disruption of such processes in FTLD. Homeostatic synaptic plasticity has been linked to neurodegeneration, possibly with loss of function due to pathogenesis, or through an increase as a mechanism to preserve function despite neurodegenerative deficits [21]. There is a known link between RNA granule formation and synapse plasticity; with RNA-binding protein function known to be particularly important. This has been proposed to be dysregulated in FTLD, whereby mutations in the genes encoding for TDP-43 and FUS lead to dysregulated granule formation dynamics and consequent disturbances in mRNA translation and synaptic function [46, 71]. Moreover, the levels of known OTUD4 protein interactor FMRP are regulated by ubiquitination in response to stimulation by the metabotropic glutamate receptor [32, 59], and this is involved in the regulation of synaptic plasticity, providing another possible link between separate findings in our study.

The *NFATC1* gene, which was also identified as an FTLD-associated loci in the EWAS meta-analysis, encodes the nuclear factor of activated T cells 1, and belongs to the NFAT family of activity-dependent transcription factors. In the nervous system, the NFAT family has been shown to play a regulatory role in neuronal excitability, axonal growth, synaptic plasticity, and neuronal survival [81]. Aberrant NFAT-related signalling has been reported in AD, and NFAT1 seems to be selectively activated early in cognitive decline [1], supporting its possible involvement in the pathogenesis of neurodegenerative diseases/dementias.

As is the case with any other genome-wide DNA methylation study, there are key limitations. First, by studying post-mortem tissue, i.e. the end stage of the disease, causality cannot be elucidated. Second, because FTLD is heterogeneous, comprising several pathological subgroups and subtypes, and given the relatively small sample size per subtype, this might have hampered the identification of additional DNA methylation alterations, especially subtype-specific variation. Notwithstanding, we focussed on the shared DNA methylation variation across FTLD subgroups/subtypes, and we used independent and complementary analytical approaches (EWAS followed by meta-analysis, and co-methylation network analysis followed by preservation analysis) and datasets, which identified concordant results and consistently identified the involvement of *OTUD4* and related genes in FTLD. Given our findings, *OTUD4* and other top hits from our meta-analysis warrant more detailed investigation in the future studies.

In summary, this study identified genome-wide DNA methylation changes in post-mortem frontal cortex tissue of FTLD subjects, highlighting new FTLD-associated loci, and implicated DNA methylation as a mechanism involved in the dysregulation of important processes such as ubiquitin and glutamatergic signalling in FTLD. Our findings increase the understanding of the biology of FTLD and role of DNA methylation its pathophysiology, pointing towards new avenues that could be explored for therapeutic development.

## Supplementary Information

Below is the link to the electronic supplementary material.

Supplementary file 1 (PDF 2726 kb)

Supplementary file 2 (XLSX 1118 kb)

## Data Availability

Raw DNA methylation data from cohorts FTLD2 and FTLD3 can be accessed via the EMBL-EBI ArrayExpress platform (accession number E-MTAB-12674) and NCBI GEO database (accession number GSE75704), respectively. Raw FTLD-TDP RNA-seq data can be accessed via the NCBI GEO database (accession number GSE153960). Additional data is available in the supplementary materials and from the corresponding author upon reasonable request.

## References

[1] Abdul HM, Furman JL, Sama MA, Mathis DM, Norris CM (2010) NFATs and Alzheimer’s disease. Mol Cell Pharmacol 2:7. 10.4255/mcpharmacol.10.02 20401186 PMC2855852

[2] Aldana BI, Zhang Y, Jensen P, Chandrasekaran A, Christensen SK, Nielsen TT et al (2020) Glutamate-glutamine homeostasis is perturbed in neurons and astrocytes derived from patient iPSC models of frontotemporal dementia. Mol Brain 13:1–17. 10.1186/S13041-020-00658-6/FIGURES/4 32928252 10.1186/s13041-020-00658-6 PMC7491073

[3] Allen M, Wang X, Serie DJ, Strickland SL, Burgess JD, Koga S et al (2018) Divergent brain gene expression patterns associate with distinct cell-specific tau neuropathology traits in progressive supranuclear palsy. Acta Neuropathol 136:709. 10.1007/S00401-018-1900-5 30136084 10.1007/s00401-018-1900-5 PMC6208732

[4] Aryee MJ, Jaffe AE, Corrada-Bravo H, Ladd-Acosta C, Feinberg AP, Hansen KD et al (2014) Minfi: a flexible and comprehensive bioconductor package for the analysis of Infinium DNA methylation microarrays. Bioinformatics. 10.1093/bioinformatics/btu049 24478339 10.1093/bioinformatics/btu049 PMC4016708

[5] Ash PEA, Lei S, Shattuck J, Boudeau S, Carlomagno Y, Medalla M et al (2021) TIA1 potentiates tau phase separation and promotes generation of toxic oligomeric tau. Proc Natl Acad Sci USA 118:e2014188118. 10.1073/PNAS.2014188118/SUPPL_FILE/PNAS.2014188118.SAPP.PDF 33619090 10.1073/pnas.2014188118 PMC7936275

[6] Aulas A, Stabile S, Vande Velde C (2012) Endogenous TDP-43, but not FUS, contributes to stress granule assembly via G3BP. Mol Neurodegener. 10.1186/1750-1326-7-54 23092511 10.1186/1750-1326-7-54 PMC3502460

[7] Bakken TE, Jorstad NL, Hu Q, Lake BB, Tian W, Kalmbach BE et al (2021) Comparative cellular analysis of motor cortex in human, marmoset and mouse. Nature 598(7879):111–119. 10.1038/s41586-021-03465-8 34616062 10.1038/s41586-021-03465-8 PMC8494640

[8] Balduzzi S, Rücker G, Schwarzer G (2019) Statistics in practice How to perform a meta-analysis with R: a practical tutorial. Evid Based Ment Health 22:153–160. 10.1136/ebmental-2019-300117 31563865 10.1136/ebmental-2019-300117 PMC10231495

[9] Banzhaf-Strathmann J, Claus R, Mücke O, Rentzsch K, van der Zee J, Engelborghs S et al (2013) Promoter DNA methylation regulates progranulin expression and is altered in FTLD. Acta Neuropathol Commun 1:1–15. 10.1186/2051-5960-1-16/FIGURES/6 10.1186/2051-5960-1-16 PMC3893557 24252647

[10] Baradaran-Heravi Y, van Broeckhoven C, van der Zee J (2020) Stress granule mediated protein aggregation and underlying gene defects in the FTD-ALS spectrum. Neurobiol Dis. 10.1016/J.NBD.2019.104639 31626953 10.1016/j.nbd.2019.104639

[11] Bettencourt C, Foti SC, Miki Y, Botia J, Chatterjee A, Warner TT et al (2020) White matter DNA methylation profiling reveals deregulation of HIP1, LMAN2, MOBP, and other loci in multiple system atrophy. Acta Neuropathol 139:135. 10.1007/S00401-019-02074-0 31535203 10.1007/s00401-019-02074-0 PMC6942018

[12] Bliss TVP, Collingridge GL (1993) A synaptic model of memory: long-term potentiation in the hippocampus. Nature 361(1993):31–39 8421494 10.1038/361031a0

[13] Botía JA, Vandrovcova J, Forabosco P, Guelfi S, D’Sa K, Hardy J et al (2017) An additional k-means clustering step improves the biological features of WGCNA gene co-expression networks. BMC Syst Biol. 10.1186/s12918-017-0420-6 28403906 10.1186/s12918-017-0420-6 PMC5389000

[14] Bowles KR, Silva MC, Whitney K, Bertucci T, Garza JC, Boles NC et al (2021) Glutamatergic dysfunction precedes neuron loss in cerebral organoids with MAPT mutation. bioRxiv. 10.1101/2021.02.03.429623 10.1016/j.cell.2021.07.003 PMC8635409 34314701

[15] Brown AL, Wilkins OG, Keuss MJ, Hill SE, Zanovello M, Lee WC et al (2022) TDP-43 loss and ALS-risk SNPs drive mis-splicing and depletion of UNC13A. Nature 603(7899):131–137. 10.1038/s41586-022-04436-3 35197628 10.1038/s41586-022-04436-3 PMC8891020

[16] Buchan JR, Parker R (2009) Eukaryotic stress granules: the ins and outs of translation. Mol Cell 36:932–941. 10.1016/J.MOLCEL.2009.11.020 20064460 10.1016/j.molcel.2009.11.020 PMC2813218

[17] Cairns NJ, Bigio EH, Mackenzie IRA, Neumann M, Lee VMY, Hatanpaa KJ et al (2007) Neuropathologic diagnostic and nosologic criteria for frontotemporal lobar degeneration: consensus of the Consortium for Frontotemporal Lobar Degeneration. Acta Neuropathol 114:5–22. 10.1007/S00401-007-0237-2/FIGURES/14 17579875 10.1007/s00401-007-0237-2 PMC2827877

[18] Campos-Melo D, Hawley ZCE, Droppelmann CA, Strong MJ (2021) The integral role of RNA in stress granule formation and function. Front Cell Dev Biol 9:808. 10.3389/FCELL.2021.621779/BIBTEX 10.3389/fcell.2021.621779 PMC8173143 34095105

[19] Chen JA, Chen Z, Won H, Huang AY, Lowe JK, Wojta K et al (2018) Joint genome-wide association study of progressive supranuclear palsy identifies novel susceptibility loci and genetic correlation to neurodegenerative diseases. Mol Neurodegener 13:1–11. 10.1186/S13024-018-0270-8/FIGURES/3 30089514 10.1186/s13024-018-0270-8 PMC6083608

[20] Das R, Schwintzer L, Vinopal S, Roca EA, Sylvester M, Oprisoreanu AM et al (2019) New roles for the de-ubiquitylating enzyme OTUD4 in an RNA-protein network and RNA granules. J Cell Sci. 10.1242/JCS.229252/VIDEO-2 31138677 10.1242/jcs.229252 PMC6602300

[21] de Pasquale R, Pignatelli M, Parsons MP, Jeans AF, Taylor HBC (2021) Friend or Foe? The varied faces of homeostatic synaptic plasticity in neurodegenerative disease. Front Cell Neurosci. 10.3389/fncel.2021.782768 10.3389/fncel.2021.782768 PMC8702499 34955753

[22] Dempster EL, Pidsley R, Schalkwyk LC, Owens S, Georgiades A, Kane F et al (2011) Disease-associated epigenetic changes in monozygotic twins discordant for schizophrenia and bipolar disorder. Hum Mol Genet 20:4786–4796. 10.1093/HMG/DDR416 21908516 10.1093/hmg/ddr416 PMC3221539

[23] Du P, Zhang X, Huang CC, Jafari N, Kibbe WA, Hou L et al (2010) Comparison of Beta-value and *M*-value methods for quantifying methylation levels by microarray analysis. BMC Bioinform. 10.1186/1471-2105-11-587 10.1186/1471-2105-11-587 PMC3012676 21118553

[24] Ferrari R, Hernandez DG, Nalls MA, Rohrer JD, Ramasamy A, Kwok JBJ et al (2014) Frontotemporal dementia and its subtypes: a genome-wide association study. Lancet Neurol 13:686–699. 10.1016/S1474-4422(14)70065-1 24943344 10.1016/S1474-4422(14)70065-1 PMC4112126

[25] Ferrer I (1999) Neurons and their dendrites in frontotemporal dementia. Dement Geriatr Cogn Disord 10(Suppl 1):55–60. 10.1159/000051214 10436342 10.1159/000051214

[26] Fyfe I (2022) Surprise neurovascular dysfunction in frontotemporal dementia. Nat Rev Neurol 18(9):509–509. 10.1038/s41582-022-00705-x 35945458 10.1038/s41582-022-00705-x

[27] Gerrits E, Giannini LAA, Brouwer N, Melhem S, Seilhean D, le Ber I et al (2022) Neurovascular dysfunction in GRN-associated frontotemporal dementia identified by single-nucleus RNA sequencing of human cerebral cortex. Nat Neurosci 25(8):1034–1048. 10.1038/s41593-022-01124-3 35879464 10.1038/s41593-022-01124-3

[28] Goldman JS, Farmer JM, Wood EM, Johnson JK, Boxer A, Neuhaus J et al (2005) Comparison of family histories in FTLD subtypes and related tauopathies. Neurology 65:1817–1819. 10.1212/01.WNL.0000187068.92184.63 16344531 10.1212/01.wnl.0000187068.92184.63

[29] Grundman J, Spencer B, Sarsoza F, Rissman RA (2021) Transcriptome analyses reveal tau isoform-driven changes in transposable element and gene expression. PLoS ONE 16:e0251611. 10.1371/journal.pone.0251611 34587152 10.1371/journal.pone.0251611 PMC8480850

[30] Haapasalo A, Vermeiren Y, Linden R, Borroni B, Benussi A, Alberici A et al (2019) Toward a glutamate hypothesis of frontotemporal dementia. Front Neurosci. 10.3389/fnins.2019.00304 30983965 10.3389/fnins.2019.00304 PMC6449454

[31] Hasan R, Humphrey J, Bettencourt C, Newcombe J, Lashley T, Fratta P et al (2022) Transcriptomic analysis of frontotemporal lobar degeneration with TDP-43 pathology reveals cellular alterations across multiple brain regions. Acta Neuropathol 143:383–401. 10.1007/S00401-021-02399-9/FIGURES/4 34961893 10.1007/s00401-021-02399-9 PMC10725322

[32] Hou L, Antion MD, Hu D, Spencer CM, Paylor R, Klann E (2006) Dynamic translational and proteasomal regulation of fragile X mental retardation protein controls mGluR-dependent long-term depression. Neuron 51:441–454. 10.1016/J.NEURON.2006.07.005 16908410 10.1016/j.neuron.2006.07.005

[33] Huang C, Cheng H, Hao S, Zhou H, Zhang X, Gao J et al (2006) Heat shock protein 70 inhibits alpha-synuclein fibril formation via interactions with diverse intermediates. J Mol Biol 364:323–336. 10.1016/J.JMB.2006.08.062 17010992 10.1016/j.jmb.2006.08.062

[34] Huber N, Korhonen S, Hoffmann D, Leskelä S, Rostalski H, Remes AM et al (2021) Deficient neurotransmitter systems and synaptic function in frontotemporal lobar degeneration—insights into disease mechanisms and current therapeutic approaches. Mol Psychiatry 27(3):1300–1309. 10.1038/s41380-021-01384-8 34799692 10.1038/s41380-021-01384-8 PMC9095474

[35] Kouri N, Ross OA, Dombroski B, Younkin CS, Serie DJ, Soto-Ortolaza A et al (2015) Genome-wide association study of corticobasal degeneration identifies risk variants shared with progressive supranuclear palsy. Nat Commun. 10.1038/NCOMMS8247 26077951 10.1038/ncomms8247 PMC4469997

[36] Krause LJ, Herrera MG, Winklhofer KF (2022) The role of ubiquitin in regulating stress granule dynamics. Front Physiol. 10.3389/FPHYS.2022.910759 35694405 10.3389/fphys.2022.910759 PMC9174786

[37] Krause LJ, Herrera MG, Winklhofer KF (2022) The role of ubiquitin in regulating stress granule dynamics. Front Physiol 13:1043. 10.3389/FPHYS.2022.910759/BIBTEX 10.3389/fphys.2022.910759 PMC9174786 35694405

[38] Krichevsky AM, Kosik KS (2001) Neuronal RNA granules: a link between RNA localization and stimulation-dependent translation. Neuron 32:683–696. 10.1016/S0896-6273(01)00508-6 11719208 10.1016/s0896-6273(01)00508-6

[39] Langfelder P, Horvath S (2008) WGCNA: an R package for weighted correlation network analysis. BMC Bioinform. 10.1186/1471-2105-9-559 10.1186/1471-2105-9-559 PMC2631488 19114008

[40] Langfelder P, Luo R, Oldham MC, Horvath S (2011) Is my network module preserved and reproducible? PLoS Comput Biol 7:1001057. 10.1371/journal.pcbi.1001057 10.1371/journal.pcbi.1001057 PMC3024255 21283776

[41] Lashley T, Rohrer JD, Bandopadhyay R, Fry C, Ahmed Z, Isaacs AM et al (2011) A comparative clinical, pathological, biochemical and genetic study of fused in sarcoma proteinopathies. Brain 134:2548–2564. 10.1093/BRAIN/AWR160 21752791 10.1093/brain/awr160 PMC3170529

[42] Lashley T, Rohrer JD, Mead S, Revesz T (2015) Review: An update on clinical, genetic and pathological aspects of frontotemporal lobar degenerations. Neuropathol Appl Neurobiol 41:858–881. 10.1111/NAN.12250 26041104 10.1111/nan.12250

[43] Leek JT, Johnson WE, Parker HS, Jaffe AE, Storey JD (2012) The SVA package for removing batch effects and other unwanted variation in high-throughput experiments. Bioinformatics. 10.1093/bioinformatics/bts034 22257669 10.1093/bioinformatics/bts034 PMC3307112

[44] Lein ES, Hawrylycz MJ, Ao N, Ayres M, Bensinger A, Bernard A et al (2006) Genome-wide atlas of gene expression in the adult mouse brain. Nature 445(7124):168–176. 10.1038/nature05453 17151600 10.1038/nature05453

[45] Liang J, Xia L, Oyang L, Lin J, Tan S, Yi P et al (2020) The functions and mechanisms of prefoldin complex and prefoldin-subunits. Cell Biosci 10:1–15. 10.1186/S13578-020-00446-8/FIGURES/6 32699605 10.1186/s13578-020-00446-8 PMC7370476

[46] Ling S-C (2018) Synaptic paths to neurodegeneration: the emerging role of TDP-43 and FUS in synaptic functions. Neural Plast. 10.1155/2018/8413496 29755516 10.1155/2018/8413496 PMC5925147

[47] Luk KC, Mills IP, Trojanowski JQ, Lee VMY (2008) Interactions between Hsp70 and the hydrophobic core of alpha-synuclein inhibit fibril assembly. Biochemistry 47:12614–12625. 10.1021/BI801475R 18975920 10.1021/bi801475r PMC2648307

[48] Lum LS, Sultzman LA, Kaufman RJ, Linzer DI, Wu BJ (1990) A cloned human CCAAT-box-binding factor stimulates transcription from the human hsp70 promoter. Mol Cell Biol 10:6709–6717. 10.1128/MCB.10.12.6709-6717.1990 2247079 10.1128/mcb.10.12.6709-6717.1990 PMC362949

[49] Margolin DH, Kousi M, Chan Y-M, Lim ET, Schmahmann JD, Hall JE et al (2013) Ataxia, dementia, and hypogonadotropism caused by disordered ubiquitination. N Engl J Med 368:1992–2003. 10.1056/NEJMoa1215993 23656588 10.1056/NEJMoa1215993 PMC3738065

[50] Mateju D, Franzmann TM, Patel A, Kopach A, Boczek EE, Maharana S et al (2017) An aberrant phase transition of stress granules triggered by misfolded protein and prevented by chaperone function. EMBO J 36:1669–1687. 10.15252/EMBJ.201695957 28377462 10.15252/embj.201695957 PMC5470046

[51] Mathys H, Davila-Velderrain J, Peng Z, Gao F, Mohammadi S, Young JZ et al (2019) Single-cell transcriptomic analysis of Alzheimer’s disease. Nature. 10.1038/s41586-019-1195-2 31042697 10.1038/s41586-019-1195-2 PMC6865822

[52] McMillan CT, Russ J, Wood EM, Irwin DJ, Grossman M, McCluskey L et al (2015) C9orf72 promoter hypermethylation is neuroprotective. Neurology 84:1622–1630. 10.1212/WNL.0000000000001495 25795648 10.1212/WNL.0000000000001495 PMC4409587

[53] Meadows JP, Guzman-Karlsson MC, Phillips S, Holleman C, Posey JL, Day JJ et al (2015) DNA methylation regulates neuronal glutamatergic synaptic scaling. Sci Signal 8:ra61. 10.1126/SCISIGNAL.AAB0715 26106219 10.1126/scisignal.aab0715 PMC4764068

[54] Menden K, Francescatto M, Nyima T, Blauwendraat C, Dhingra A, Castillo-Lizardo M et al (2022) A multi-omics dataset for the analysis of frontotemporal dementia genetic subtypes. bioRxiv. 10.1101/2020.12.01.405894 10.1038/s41597-023-02598-x PMC10692098 38040703

[55] Mevissen TET, Hospenthal MK, Geurink PP, Elliott PR, Akutsu M, Arnaudo N et al (2013) OTU deubiquitinases reveal mechanisms of linkage specificity and enable ubiquitin chain restriction analysis. Cell 154:169. 10.1016/J.CELL.2013.05.046 23827681 10.1016/j.cell.2013.05.046 PMC3705208

[56] Müller S (2021) Managing stress granule disassembly with ubiquitin and its cousin. Signal Transduct Targeted Ther 6(1):1–2. 10.1038/s41392-021-00782-2 10.1038/s41392-021-00782-2 PMC8586141 34764249

[57] Murley AG, Rowe JB (2018) Neurotransmitter deficits from frontotemporal lobar degeneration. Brain 141:1263–1285. 10.1093/BRAIN/AWX327 29373632 10.1093/brain/awx327 PMC5917782

[58] Murray CE, Gami-Patel P, Gkanatsiou E, Brinkmalm G, Portelius E, Wirths O et al (2018) The presubiculum is preserved from neurodegenerative changes in Alzheimer’s disease. Acta Neuropathol Commun 6:1–17. 10.1186/S40478-018-0563-8/FIGURES/7 30029687 10.1186/s40478-018-0563-8 PMC6053705

[59] Nalavadi VC, Muddashetty RS, Gross C, Bassell GJ (2012) Dephosphorylation-induced ubiquitination and degradation of FMRP in dendrites: a role in immediate early mGluR-stimulated translation. J Neurosci 32:2582–2587. 10.1523/JNEUROSCI.5057-11.2012 22357842 10.1523/JNEUROSCI.5057-11.2012 PMC3427762

[60] Neumann M, Sampathu DM, Kwong LK, Truax AC, Micsenyi MC, Chou TT et al (2006) Ubiquitinated TDP-43 in frontotemporal lobar degeneration and amyotrophic lateral sclerosis. Science 314:130–133. 10.1126/SCIENCE.1134108 17023659 10.1126/science.1134108

[61] Pai AA, Bell JT, Marioni JC, Pritchard JK, Gilad Y (2011) A genome-wide study of DNA methylation patterns and gene expression levels in multiple human and chimpanzee tissues. PLoS Genet 7:1001316. 10.1371/JOURNAL.PGEN.1001316 10.1371/journal.pgen.1001316 PMC3044686 21383968

[62] Pedrola L, Espert A, Wu X, Claramunt R, Shy ME, Palau F (2005) GDAP1, the protein causing Charcot-Marie-Tooth disease type 4A, is expressed in neurons and is associated with mitochondria. Hum Mol Genet 14:1087–1094. 10.1093/hmg/ddi121 15772096 10.1093/hmg/ddi121

[63] Pemberton S, Madiona K, Pieri L, Kabani M, Bousset L, Melki R (2011) Hsc70 protein interaction with soluble and fibrillar alpha-synuclein. J Biol Chem 286:34690–34699. 10.1074/JBC.M111.261321 21832061 10.1074/jbc.M111.261321 PMC3186418

[64] Pottier C, Mateiu L, Baker MC, Dejesus-Hernandez M, Teixeira Vicente C, Finch NA et al (2022) Shared brain transcriptomic signature in TDP-43 type A FTLD patients with or without GRN mutations. Brain 145:2472–2485. 10.1093/BRAIN/AWAB437 34918030 10.1093/brain/awab437 PMC9337811

[65] Ritchie ME, Phipson B, Wu D, Hu Y, Law CW, Shi W et al (2015) limma powers differential expression analyses for RNA-sequencing and microarray studies. Nucleic Acids Res 43:e47–e47. 10.1093/NAR/GKV007 25605792 10.1093/nar/gkv007 PMC4402510

[66] Rouillard AD, Gundersen GW, Fernandez NF, Wang Z, Monteiro CD, McDermott MG et al (2016) The harmonizome: a collection of processed datasets gathered to serve and mine knowledge about genes and proteins. Database. 10.1093/DATABASE/BAW100 27374120 10.1093/database/baw100 PMC4930834

[67] Sanchez-Contreras MY, Kouri N, Cook CN, Serie DJ, Heckman MG, Finch NA et al (2018) Replication of progressive supranuclear palsy genome-wide association study identifies SLCO1A2 and DUSP10 as new susceptibility loci. Mol Neurodegener 13:1–10. 10.1186/S13024-018-0267-3/TABLES/4 29986742 10.1186/s13024-018-0267-3 PMC6038352

[68] Schalkwyk LC, Pidsley R, Wong CCY (2013) wateRmelon: illumina 450 methylation array normalization and metrics. R Package Version 1(2):2

[69] Schmidt MF, Gan ZY, Komander D, Dewson G (2021) Ubiquitin signalling in neurodegeneration: mechanisms and therapeutic opportunities. Cell Death Differ 28(2):570–590. 10.1038/s41418-020-00706-7 33414510 10.1038/s41418-020-00706-7 PMC7862249

[70] Schofield E, Kersaitis C, Shepherd CE, Kril JJ, Halliday GM (2003) Severity of gliosis in Pick’s disease and frontotemporal lobar degeneration: tau-positive glia differentiate these disorders. Brain 126:827–840. 10.1093/BRAIN/AWG085 12615642 10.1093/brain/awg085

[71] Sephton CF, Yu G (2015) The function of RNA-binding proteins at the synapse: implications for neurodegeneration. Cell Mol Life Sci 72:3621. 10.1007/S00018-015-1943-X 26047658 10.1007/s00018-015-1943-x PMC4565867

[72] Sethna F, Feng W, Ding Q, Robison AJ, Feng Y, Wang H (2017) Enhanced expression of ADCY1 underlies aberrant neuronal signalling and behaviour in a syndromic autism model. Nat Commun. 10.1038/NCOMMS14359 28218269 10.1038/ncomms14359 PMC5321753

[73] Shireby G, Dempster EL, Policicchio S, Smith RG, Pishva E, Chioza B et al (2022) DNA methylation signatures of Alzheimer’s disease neuropathology in the cortex are primarily driven by variation in non-neuronal cell-types. Nat Commun 13(1):1–14. 10.1038/s41467-022-33394-7 36153390 10.1038/s41467-022-33394-7 PMC9509387

[74] Skene NG, Grant SGN (2016) Identification of vulnerable cell types in major brain disorders using single cell transcriptomes and expression weighted cell type enrichment. Front Neurosci 10:16. 10.3389/FNINS.2016.00016/BIBTEX 26858593 10.3389/fnins.2016.00016 PMC4730103

[75] Smith RG, Pishva E, Shireby G, Smith AR, Roubroeks JAY, Hannon E et al (2021) A meta-analysis of epigenome-wide association studies in Alzheimer’s disease highlights novel differentially methylated loci across cortex. Nat Commun 12(1):1–13. 10.1038/s41467-021-23243-4 34112773 10.1038/s41467-021-23243-4 PMC8192929

[76] Sörgjerd KM, Zako T, Sakono M, Stirling PC, Leroux MR, Saito T et al (2013) Human prefoldin inhibits amyloid-β (Aβ) fibrillation and contributes to formation of nontoxic Aβ aggregates. Biochemistry 52:3532–3542. 10.1021/BI301705C 23614719 10.1021/bi301705c

[77] Tian Y, Morris TJ, Webster AP, Yang Z, Beck S, Feber A et al (2017) ChAMP: Updated methylation analysis pipeline for Illumina BeadChips. Bioinformatics. 10.1093/bioinformatics/btx513 28961746 10.1093/bioinformatics/btx513 PMC5860089

[78] Tolay N, Buchberger A (2022) Role of the ubiquitin system in stress granule metabolism. Int J Mol Sci 23:3624. 10.3390/IJMS23073624 35408984 10.3390/ijms23073624 PMC8999021

[79] Toomey CE, Heywood W, Benson BC, Packham G, Mills K, Lashley T (2020) Investigation of pathology, expression and proteomic profiles in human TREM2 variant postmortem brains with and without Alzheimer’s disease. Brain Pathol 30:794–810. 10.1111/BPA.12842 32267026 10.1111/bpa.12842 PMC8018003

[80] Vanderkraats ND, Hiken JF, Decker KF, Edwards JR (2013) Discovering high-resolution patterns of differential DNA methylation that correlate with gene expression changes. Nucl Acids Res 41:6816–6827. 10.1093/NAR/GKT482 23748561 10.1093/nar/gkt482 PMC3737560

[81] Vihma H, Luhakooder M, Pruunsild P, Timmusk T (2016) Regulation of different human NFAT isoforms by neuronal activity. J Neurochem 137:394–408. 10.1111/JNC.13568 26851544 10.1111/jnc.13568

[82] Wang X, Allen M, İş Ö, Reddy JS, Tutor-New FQ, Casey MC et al (2022) Alzheimer’s disease and progressive supranuclear palsy share similar transcriptomic changes in distinct brain regions. J Clin Invest. 10.1172/JCI149904 34813500 10.1172/JCI149904 PMC8759790

[83] Wang X, Allen M, Reddy JS, Carrasquillo MM, Asmann YW, Funk C et al (2021) Conserved architecture of brain transcriptome changes between Alzheimer’s disease and progressive supranuclear palsy in pathologically affected and unaffected regions. bioRxiv. 10.1101/2021.01.18.42699935018379

[84] Weber A, Schwarz SC, Tost J, Trümbach D, Winter P, Busato F et al (2018) Epigenome-wide DNA methylation profiling in Progressive Supranuclear Palsy reveals major changes at DLX1. Nat Commun. 10.1038/s41467-018-05325-y 30050033 10.1038/s41467-018-05325-y PMC6062504

[85] Williams DR, Holton JL, Strand C, Pittman A, de Silva R, Lees AJ et al (2007) Pathological tau burden and distribution distinguishes progressive supranuclear palsy-parkinsonism from Richardson’s syndrome. Brain 130:1566–1576. 10.1093/BRAIN/AWM104 17525140 10.1093/brain/awm104

[86] Wu T, Hu E, Xu S, Chen M, Guo P, Dai Z et al (2021) ClusterProfiler 4.0: a universal enrichment tool for interpreting omics data. Innovation 2:100141. 10.1016/J.XINN.2021.100141 34557778 10.1016/j.xinn.2021.100141 PMC8454663

[87] Xi Z, Zhang M, Bruni AC, Maletta RG, Colao R, Fratta P et al (2015) The C9orf72 repeat expansion itself is methylated in ALS and FTLD patients. Acta Neuropathol 129:715–727. 10.1007/S00401-015-1401-8/METRICS 25716178 10.1007/s00401-015-1401-8

[88] Yokoyama JS, Karch CM, Fan CC, Bonham LW, Kouri N, Ross OA et al (2017) Shared genetic risk between corticobasal degeneration, progressive supranuclear palsy, and frontotemporal dementia. Acta Neuropathol 133:825. 10.1007/S00401-017-1693-Y 28271184 10.1007/s00401-017-1693-y PMC5429027

[89] Zeisel A, Muñoz-Manchado AB, Codeluppi S, Lönnerberg P, La MG, Juréus A et al (2015) Cell types in the mouse cortex and hippocampus revealed by single-cell RNA-seq. Science (1979) 347:1138–1142. 10.1126/SCIENCE.AAA1934 10.1126/science.aaa1934 25700174

[90] Zhang L, Silva TC, Young JI, Gomez L, Schmidt MA, Hamilton-Nelson KL et al (2020) Epigenome-wide meta-analysis of DNA methylation differences in prefrontal cortex implicates the immune processes in Alzheimer’s disease. Nat Commun 11(1):1–13. 10.1038/s41467-020-19791-w 33257653 10.1038/s41467-020-19791-w PMC7704686

[91] Zhao Y, Majid MC, Soll JM, Brickner JR, Dango S, Mosammaparast N (2015) Noncanonical regulation of alkylation damage resistance by the OTUD4 deubiquitinase. EMBO J 34:1687–1703. 10.15252/EMBJ.201490497 25944111 10.15252/embj.201490497 PMC4475402

[92] Zhao Y, Mudge MC, Soll JM, Rodrigues RB, Byrum AK, Schwarzkopf EA et al (2018) OTUD4 is a phospho-activated K63 deubiquitinase that regulates MyD88-dependent signaling. Mol Cell 69:505-516.e5. 10.1016/j.molcel.2018.01.009 29395066 10.1016/j.molcel.2018.01.009 PMC6819006

[93] Zhou Y, Danbolt NC (2014) Glutamate as a neurotransmitter in the healthy brain. J Neural Transm 121:799. 10.1007/S00702-014-1180-8 24578174 10.1007/s00702-014-1180-8 PMC4133642

