# Supplementary material for: Brain DNA methylomic analysis of frontotemporal lobar degeneration reveals OTUD4 in shared dysregulated signatures across pathological subtypes
Source: Acta Neuropathol. 2023 May 7;146(1):77–95. doi: 10.1007/s00401-023-02583-z (PMC10261190; doi:10.1007/s00401-023-02583-z)
Supplement: Supplementary file 1 — (PDF 2726 kb) [file 401_2023_2583_MOESM1_ESM.pdf]

Brain DNA methylomic analysis of frontotemporal lobar degeneration reveals *OTUD4* in shared dysregulated signatures across pathological subtypes

Katherine Fodder[1,2], Megha Murthy[1,3], Patrizia Rizzu[4], Christina E. Toomey[1,3,5], Rahat Hasan[6], Jack Humphrey[6], Towfique Raj[6], Katie Lunnon[7], Jonathan Mill[7], Peter Heutink[4,8], Tammaryn Lashley[1,2], Conceição Bettencourt[1,2,*]

1. Queen Square Brain Bank for Neurological Disorders, UCL Queen Square Institute of Neurology, London, UK.
2. Department of Neurodegenerative Disease, UCL Queen Square Institute of Neurology, London, UK.
3. Department of Clinical and Movement Neurosciences, UCL Queen Square Institute of Neurology, London, UK.
4. German Center for Neurodegenerative Diseases (DZNE), Tübingen, Germany.
5. The Francis Crick Institute, London, UK.
6. Nash Family Department of Neuroscience & Friedman Brain Institute, Icahn School of Medicine at Mount Sinai, New York, NY, USA.
7. Department of Clinical and Biomedical Sciences, Faculty of Health and Life Sciences, University of Exeter, Exeter, UK.
8. Alector, Inc., South San Francisco, CA, USA.

***Corresponding Author:**

Conceição Bettencourt, PhD

Department of Neurodegenerative Disease

UCL Queen Square Institute of Neurology

Queen Square Brain Bank for Neurological Disorders

1 Wakefield Street

London WC1N 1PJ

United Kingdom

Email c.bettencourt@ucl.ac.uk

a)

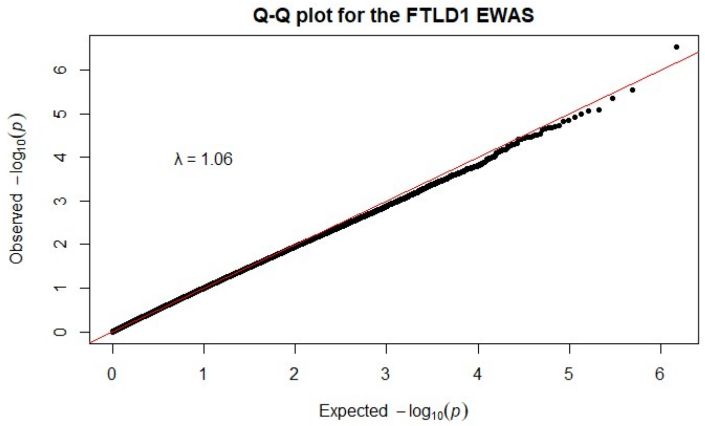

b)

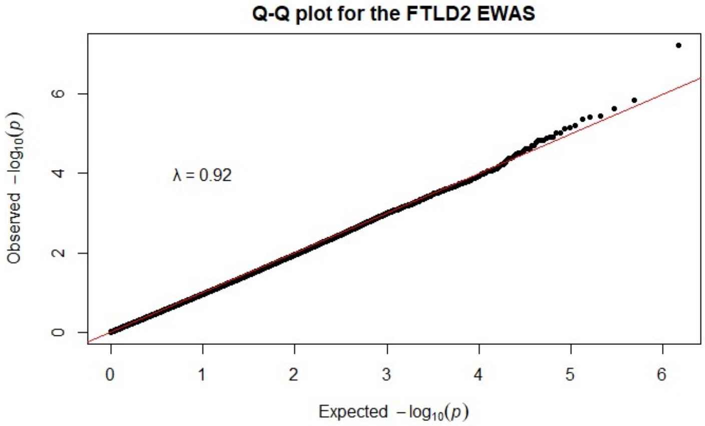

c)

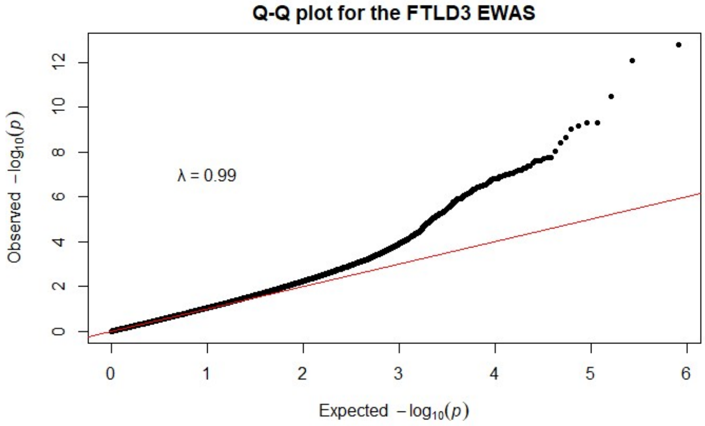

Supplementary figure 1. Quantile-quantile (Q-Q) plots for the FTLD cohort-specific case-control EWAS. a) Q-Q plot for the FTLD1 EWAS, with an estimated inflation factor (λ) of 1.06; **b)** Q-Q plot for the FTLD2 EWAS, with an estimated inflation factor (λ) of 0.92; **c)** Q-Q plot for the FTLD3 EWAS, with an estimated inflation factor (λ) of 0.99.

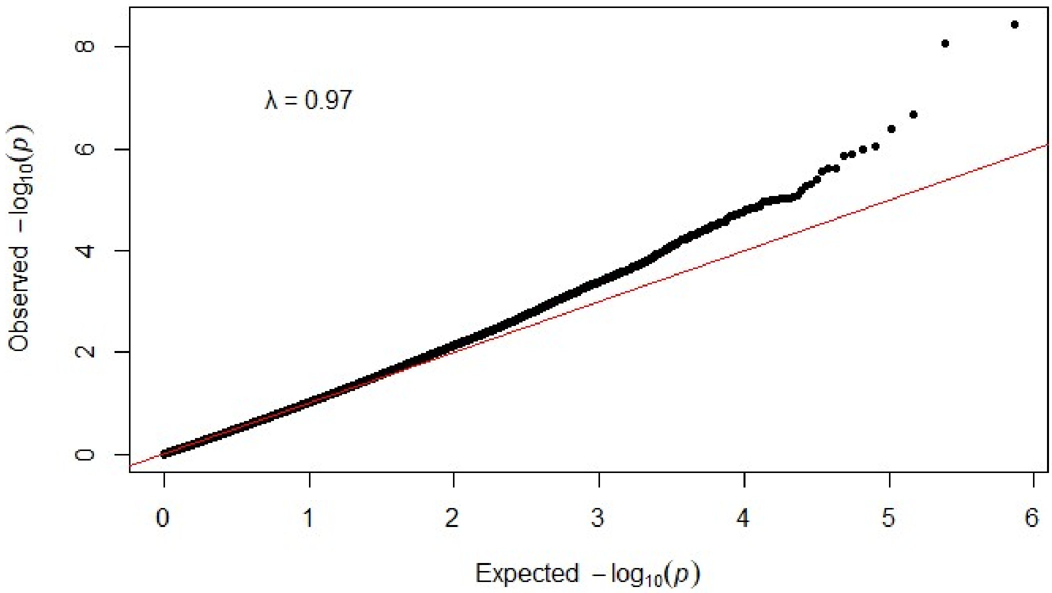

Supplementary figure 2. Quantile-quantile (Q-Q) plots for the FTLD case-control EWAS meta-analysis with random-effect models across three cohorts (FTLD1, FTLD2 and FTLD3).

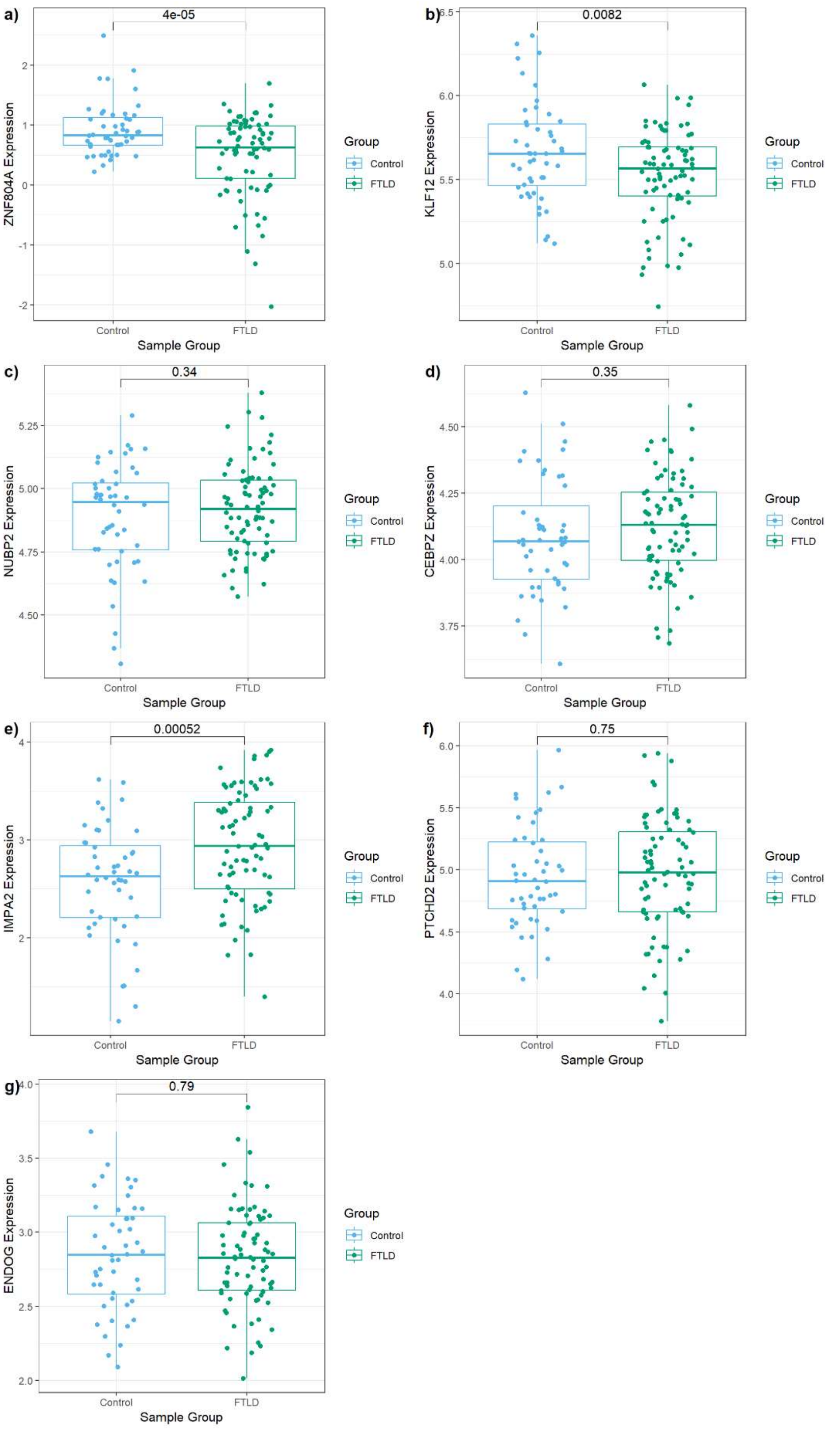

Supplementary figure 3. Boxplots showing gene expression levels for the top EWAS meta-analysis loci in FTLD-TDP and controls. RNA sequencing data from Hasan et al.[2] adjusted for age, sex, and RNA integrity number was used. Log2-transformed gene expression data is shown in the y-axis, and non-paired t-test p-value for the comparison between FTLD-TDP (N=80) and controls (N=48) is denoted at the top.

a) **Module−trait relationships (k−means) of FTLD1**

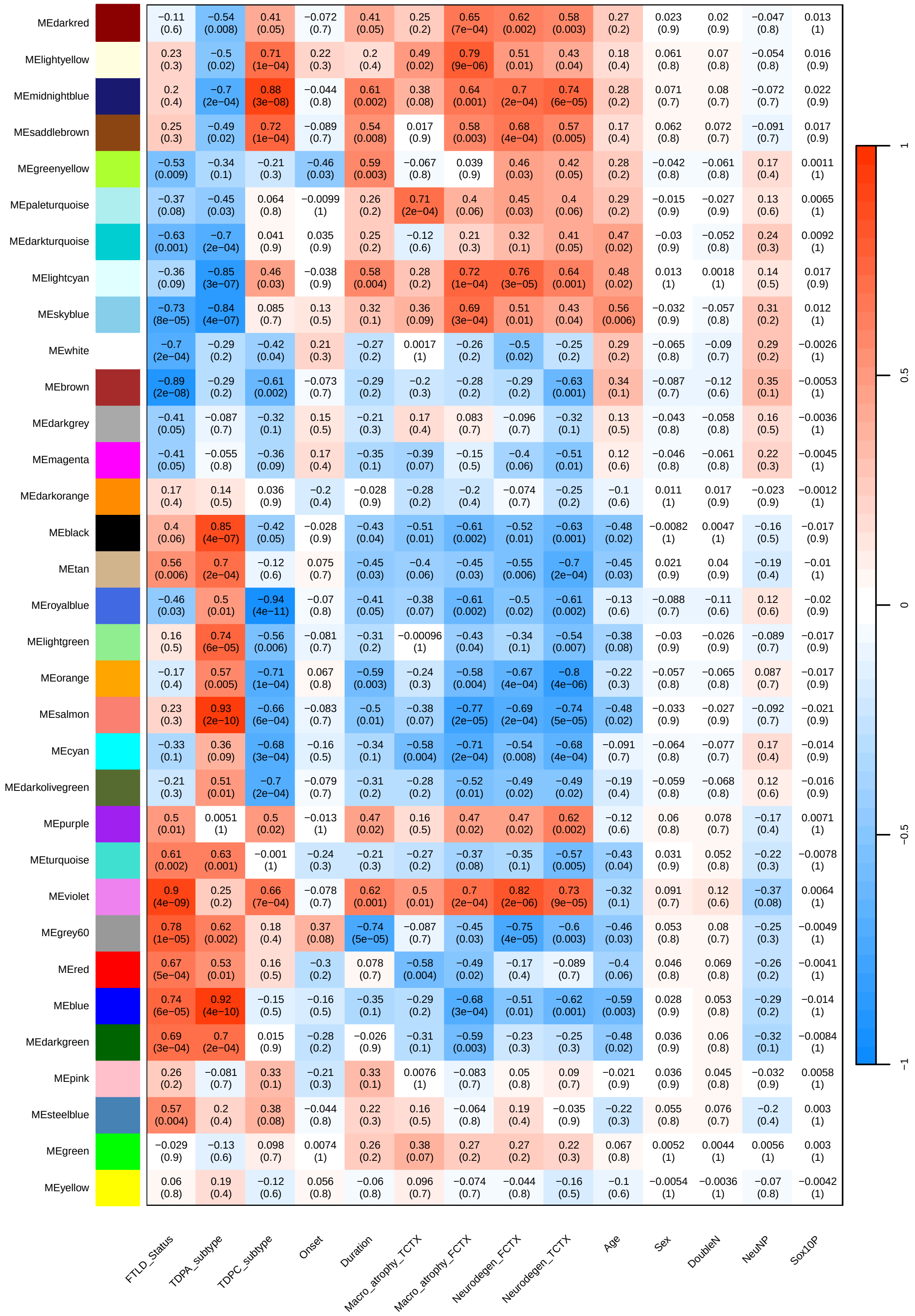

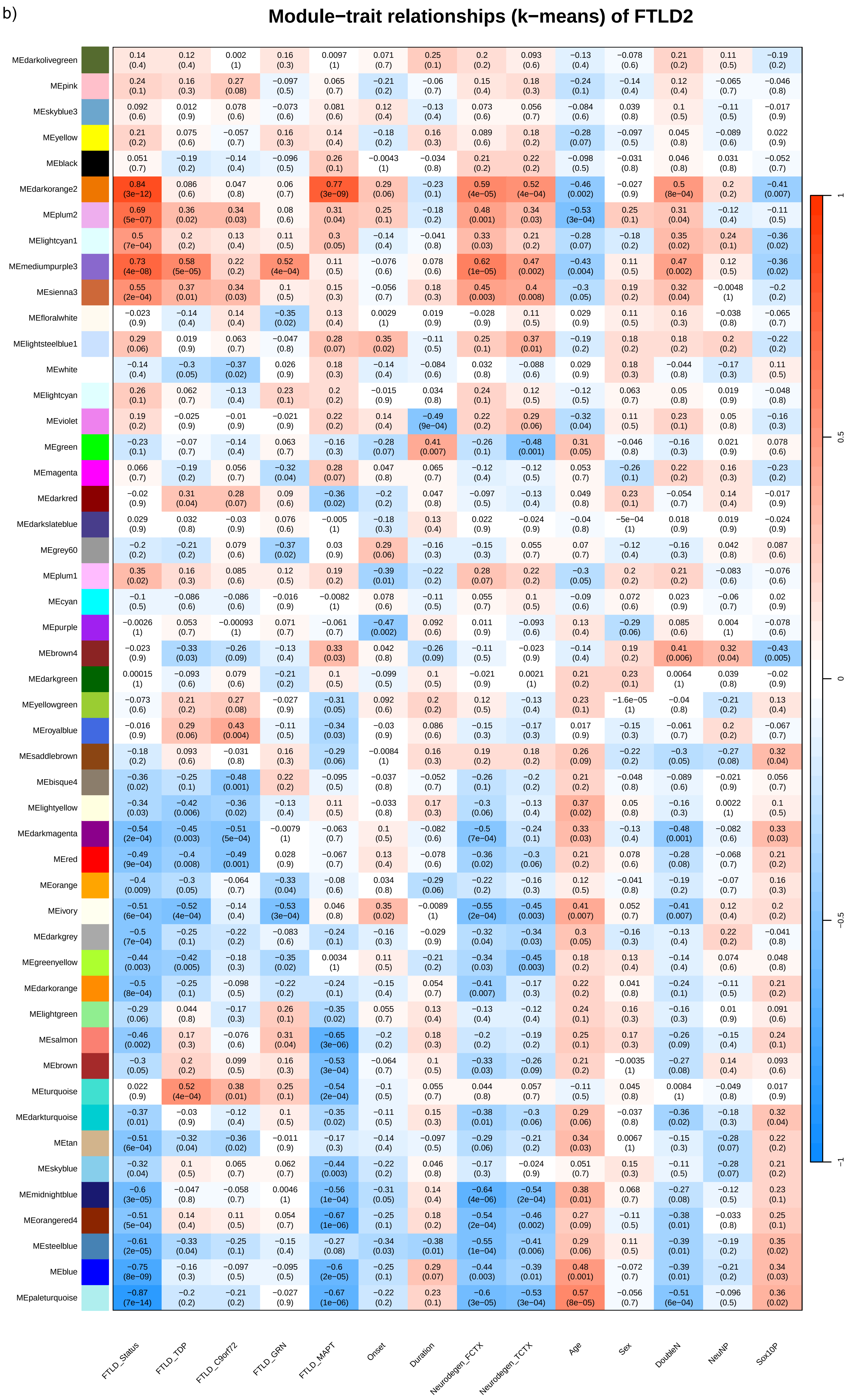
b)
Module−trait relationships (k−means) of FTLD2
MEdarkolivegreen 0.14 (0.4) 0.12 (0.4) 0.002 (1) 0.16 (0.3) 0.0097 (1) 0.071 (0.7) 0.25 (0.1) 0.2 (0.2) 0.093 (0.6) −0.13 (0.4) −0.078 (0.6) 0.21 (0.2) 0.11 (0.5) −0.19 (0.2)
MEpink 0.24 (0.1) 0.16 (0.3) 0.27 (0.08) −0.097 (0.5) 0.065 (0.7) −0.21 (0.2) −0.06 (0.7) 0.15 (0.4) 0.18 (0.3) −0.24 (0.1) −0.14 (0.4) 0.12 (0.4) −0.065 (0.7) −0.046 (0.8)
MEskyblue3 0.092 (0.6) 0.012 (0.9) 0.078 (0.6) −0.073 (0.6) 0.081 (0.6) 0.12 (0.4) −0.13 (0.4) 0.073 (0.6) 0.056 (0.7) −0.084 (0.6) 0.039 (0.8) 0.1 (0.5) −0.11 (0.5) −0.017 (0.9)
MEyellow 0.21 (0.2) 0.075 (0.6) −0.057 (0.7) 0.16 (0.3) 0.14 (0.4) −0.18 (0.2) 0.16 (0.3) 0.089 (0.6) 0.18 (0.2) −0.28 (0.07) −0.097 (0.5) 0.045 (0.8) −0.089 (0.6) 0.022 (0.9)
MEblack 0.051 (0.7) −0.19 (0.2) −0.14 (0.4) −0.096 (0.5) 0.26 (0.1) −0.0043 (1) −0.034 (0.8) 0.21 (0.2) 0.22 (0.2) −0.098 (0.5) −0.031 (0.8) 0.046 (0.8) 0.031 (0.8) −0.052 (0.7)
MEdarkorange2 0.84 (3e−12) 0.086 (0.6) 0.047 (0.8) 0.06 (0.7) 0.77 (3e−09) 0.29 (0.06) −0.23 (0.1) 0.59 (4e−05) 0.52 (4e−04) −0.46 (0.002) −0.027 (0.9) 0.5 (8e−04) 0.2 (0.2) −0.41 (0.007)
MEplum2 0.69 (5e−07) 0.36 (0.02) 0.34 (0.03) 0.08 (0.6) 0.31 (0.04) 0.25 (0.1) −0.18 (0.2) 0.48 (0.001) 0.34 (0.03) −0.53 (3e−04) 0.25 (0.1) 0.31 (0.04) −0.12 (0.4) −0.11 (0.5)
MElightcyan1 0.5 (7e−04) 0.2 (0.2) 0.13 (0.4) 0.11 (0.5) 0.3 (0.05) −0.14 (0.4) −0.041 (0.8) 0.33 (0.03) 0.21 (0.2) −0.28 (0.07) −0.18 (0.2) 0.35 (0.02) 0.24 (0.1) −0.36 (0.02)
MEmediumpurple3 0.73 (4e−08) 0.58 (5e−05) 0.22 (0.2) 0.52 (4e−04) 0.11 (0.5) −0.076 (0.6) 0.078 (0.6) 0.62 (1e−05) 0.47 (0.002) −0.43 (0.004) 0.11 (0.5) 0.47 (0.002) 0.12 (0.5) −0.36 (0.02)
MEsienna3 0.55 (2e−04) 0.37 (0.01) 0.34 (0.03) 0.1 (0.5) 0.15 (0.3) −0.056 (0.7) 0.18 (0.3) 0.45 (0.003) 0.4 (0.008) −0.3 (0.05) 0.19 (0.2) 0.32 (0.04) −0.0048 (1) −0.2 (0.2)
MEfloralwhite −0.023 (0.9) −0.14 (0.4) 0.14 (0.4) −0.35 (0.02) 0.13 (0.4) 0.0029 (1) 0.019 (0.9) −0.028 (0.9) 0.11 (0.5) 0.029 (0.9) 0.11 (0.5) 0.16 (0.3) −0.038 (0.8) −0.065 (0.7)
MElightsteelblue1 0.29 (0.06) 0.019 (0.9) 0.063 (0.7) −0.047 (0.8) 0.28 (0.07) 0.35 (0.02) −0.11 (0.5) 0.25 (0.1) 0.37 (0.01) −0.19 (0.2) 0.18 (0.2) 0.18 (0.2) 0.2 (0.2) −0.22 (0.2)
MEwhite −0.14 (0.4) −0.3 (0.05) −0.37 (0.02) 0.026 (0.9) 0.18 (0.3) −0.14 (0.4) −0.084 (0.6) 0.032 (0.8) −0.088 (0.6) 0.029 (0.9) 0.18 (0.3) −0.044 (0.8) −0.17 (0.3) 0.11 (0.5)
MElightcyan 0.26 (0.1) 0.062 (0.7) −0.13 (0.4) 0.23 (0.1) 0.2 (0.2) −0.015 (0.9) 0.034 (0.8) 0.24 (0.1) 0.12 (0.5) −0.12 (0.5) 0.063 (0.7) 0.05 (0.8) 0.019 (0.9) −0.048 (0.8)
MEviolet 0.19 (0.2) −0.025 (0.9) −0.01 (0.9) −0.021 (0.9) 0.22 (0.2) 0.14 (0.4) −0.49 (9e−04) 0.22 (0.2) 0.29 (0.06) −0.32 (0.04) 0.11 (0.5) 0.23 (0.1) 0.05 (0.8) −0.16 (0.3)
MEgreen −0.23 (0.1) −0.07 (0.7) −0.14 (0.4) 0.063 (0.7) −0.16 (0.3) −0.28 (0.07) 0.41 (0.007) −0.26 (0.1) −0.48 (0.001) 0.31 (0.05) −0.046 (0.8) −0.16 (0.3) 0.021 (0.9) 0.078 (0.6)
MEmagenta 0.066 (0.7) −0.19 (0.2) 0.056 (0.7) −0.32 (0.04) 0.28 (0.07) 0.047 (0.8) 0.065 (0.7) −0.12 (0.4) −0.12 (0.5) 0.053 (0.7) −0.26 (0.1) 0.22 (0.2) 0.16 (0.3) −0.23 (0.2)
MEdarkred −0.02 (0.9) 0.31 (0.04) 0.28 (0.07) 0.09 (0.6) −0.36 (0.02) −0.2 (0.2) 0.047 (0.8) −0.097 (0.5) −0.13 (0.4) 0.049 (0.8) 0.23 (0.1) −0.054 (0.7) 0.14 (0.4) −0.017 (0.9)
MEdarkslateblue 0.029 (0.9) 0.032 (0.8) −0.03 (0.9) 0.076 (0.6) −0.005 (1) −0.18 (0.3) 0.13 (0.4) 0.022 (0.9) −0.024 (0.9) −0.04 (0.8) −5e−04 (1) 0.018 (0.9) 0.019 (0.9) −0.024 (0.9)
MEgrey60 −0.2 (0.2) −0.21 (0.2) 0.079 (0.6) −0.37 (0.02) 0.03 (0.9) 0.29 (0.06) −0.16 (0.3) −0.15 (0.3) 0.055 (0.7) 0.07 (0.7) −0.12 (0.4) −0.16 (0.3) 0.042 (0.8) 0.087 (0.6)
MEplum1 0.35 (0.02) 0.16 (0.3) 0.085 (0.6) 0.12 (0.5) 0.19 (0.2) −0.39 (0.01) −0.22 (0.2) 0.28 (0.07) 0.22 (0.2) −0.3 (0.05) 0.2 (0.2) 0.21 (0.2) −0.083 (0.6) −0.076 (0.6)
MEcyan −0.1 (0.5) −0.086 (0.6) −0.086 (0.6) −0.016 (0.9) −0.0082 (1) 0.078 (0.6) −0.11 (0.5) 0.055 (0.7) 0.1 (0.5) −0.09 (0.6) 0.072 (0.6) 0.023 (0.9) −0.06 (0.7) 0.02 (0.9)
MEpurple −0.0026 (1) 0.053 (0.7) −0.00093 (1) 0.071 (0.7) −0.061 (0.7) −0.47 (0.002) 0.092 (0.6) 0.011 (0.9) −0.093 (0.6) 0.13 (0.4) −0.29 (0.06) 0.085 (0.6) 0.004 (1) −0.078 (0.6)
MEbrown4 −0.023 (0.9) −0.33 (0.03) −0.26 (0.09) −0.13 (0.4) 0.33 (0.03) 0.042 (0.8) −0.26 (0.09) −0.11 (0.5) −0.023 (0.9) −0.14 (0.4) 0.19 (0.2) 0.41 (0.006) 0.32 (0.04) −0.43 (0.005)
MEdarkgreen 0.00015 (1) −0.093 (0.6) 0.079 (0.6) −0.21 (0.2) 0.1 (0.5) −0.099 (0.5) 0.1 (0.5) −0.021 (0.9) 0.0021 (1) 0.21 (0.2) 0.23 (0.1) 0.0064 (1) 0.039 (0.8) −0.02 (0.9)
MEyellowgreen −0.073 (0.6) 0.21 (0.2) 0.27 (0.08) −0.027 (0.9) −0.31 (0.05) 0.092 (0.6) 0.2 (0.2) 0.12 (0.5) −0.13 (0.4) 0.23 (0.1) −1.6e−05 (1) −0.04 (0.8) −0.21 (0.2) 0.13 (0.4)
MEroyalblue −0.016 (0.9) 0.29 (0.06) 0.43 (0.004) −0.11 (0.5) −0.34 (0.03) −0.03 (0.9) 0.086 (0.6) −0.15 (0.3) −0.17 (0.3) 0.017 (0.9) −0.15 (0.3) −0.061 (0.7) 0.2 (0.2) −0.067 (0.7)
MEsaddlebrown −0.18 (0.2) 0.093 (0.6) −0.031 (0.8) 0.16 (0.3) −0.29 (0.06) −0.0084 (1) 0.16 (0.3) 0.19 (0.2) 0.18 (0.2) 0.26 (0.09) −0.22 (0.2) −0.3 (0.05) −0.27 (0.08) 0.32 (0.04)
MEbisque4 −0.36 (0.02) −0.25 (0.1) −0.48 (0.001) 0.22 (0.2) −0.095 (0.5) −0.037 (0.8) −0.052 (0.7) −0.26 (0.1) −0.2 (0.2) 0.21 (0.2) −0.048 (0.8) −0.089 (0.6) −0.021 (0.9) 0.056 (0.7)
MElightyellow −0.34 (0.03) −0.42 (0.006) −0.36 (0.02) −0.13 (0.4) 0.11 (0.5) −0.033 (0.8) 0.17 (0.3) −0.3 (0.06) −0.13 (0.4) 0.37 (0.02) 0.05 (0.8) −0.16 (0.3) 0.0022 (1) 0.1 (0.5)
MEdarkmagenta −0.54 (2e−04) −0.45 (0.003) −0.51 (5e−04) −0.0079 (1) −0.063 (0.7) 0.1 (0.5) −0.082 (0.6) −0.5 (7e−04) −0.24 (0.1) 0.33 (0.03) −0.13 (0.4) −0.48 (0.001) −0.082 (0.6) 0.33 (0.03)
MEred −0.49 (9e−04) −0.4 (0.008) −0.49 (0.001) 0.028 (0.9) −0.067 (0.7) 0.13 (0.4) −0.078 (0.6) −0.36 (0.02) −0.3 (0.06) 0.21 (0.2) 0.078 (0.6) −0.28 (0.08) −0.068 (0.7) 0.21 (0.2)
MEorange −0.4 (0.009) −0.3 (0.05) −0.064 (0.7) −0.33 (0.04) −0.08 (0.6) 0.034 (0.8) −0.29 (0.06) −0.22 (0.2) −0.16 (0.3) 0.12 (0.5) −0.041 (0.8) −0.19 (0.2) −0.07 (0.7) 0.16 (0.3)
MEivory −0.51 (6e−04) −0.52 (4e−04) −0.14 (0.4) −0.53 (3e−04) 0.046 (0.8) 0.35 (0.02) −0.0089 (1) −0.55 (2e−04) −0.45 (0.003) 0.41 (0.007) 0.052 (0.7) −0.41 (0.007) 0.12 (0.4) 0.2 (0.2)
MEdarkgrey −0.5 (7e−04) −0.25 (0.1) −0.22 (0.2) −0.083 (0.6) −0.24 (0.1) −0.16 (0.3) −0.029 (0.9) −0.32 (0.04) −0.34 (0.03) 0.3 (0.05) −0.16 (0.3) −0.13 (0.4) 0.22 (0.2) −0.041 (0.8)
MEgreenyellow −0.44 (0.003) −0.42 (0.005) −0.18 (0.3) −0.35 (0.02) 0.0034 (1) 0.11 (0.5) −0.21 (0.2) −0.34 (0.03) −0.45 (0.003) 0.18 (0.2) 0.13 (0.4) −0.14 (0.4) 0.074 (0.6) 0.048 (0.8)
MEdarkorange −0.5 (8e−04) −0.25 (0.1) −0.098 (0.5) −0.22 (0.2) −0.24 (0.1) −0.15 (0.4) 0.054 (0.7) −0.41 (0.007) −0.17 (0.3) 0.22 (0.2) 0.041 (0.8) −0.24 (0.1) −0.11 (0.5) 0.21 (0.2)
MElightgreen −0.29 (0.06) 0.044 (0.8) −0.17 (0.3) 0.26 (0.1) −0.35 (0.02) 0.055 (0.7) 0.13 (0.4) −0.13 (0.4) −0.12 (0.4) 0.24 (0.1) 0.16 (0.3) −0.16 (0.3) 0.01 (0.9) 0.091 (0.6)
MEsalmon −0.46 (0.002) 0.17 (0.3) −0.076 (0.6) 0.31 (0.04) −0.65 (3e−06) −0.2 (0.2) 0.18 (0.3) −0.2 (0.2) −0.19 (0.2) 0.25 (0.1) 0.17 (0.3) −0.26 (0.09) −0.15 (0.4) 0.24 (0.1)
MEbrown −0.3 (0.05) 0.2 (0.2) 0.099 (0.5) 0.16 (0.3) −0.53 (3e−04) −0.064 (0.7) 0.1 (0.5) −0.33 (0.03) −0.26 (0.09) 0.21 (0.2) −0.0035 (1) −0.27 (0.08) 0.14 (0.4) 0.093 (0.6)
MEturquoise 0.022 (0.9) 0.52 (4e−04) 0.38 (0.01) 0.25 (0.1) −0.54 (2e−04) −0.1 (0.5) 0.055 (0.7) 0.044 (0.8) 0.057 (0.7) −0.11 (0.5) 0.045 (0.8) 0.0084 (1) −0.049 (0.8) 0.017 (0.9)
MEdarkturquoise −0.37 (0.01) −0.03 (0.9) −0.12 (0.4) 0.1 (0.5) −0.35 (0.02) −0.11 (0.5) 0.15 (0.3) −0.38 (0.01) −0.3 (0.06) 0.29 (0.06) −0.037 (0.8) −0.36 (0.02) −0.18 (0.3) 0.32 (0.04)
MEtan −0.51 (6e−04) −0.32 (0.04) −0.36 (0.02) −0.011 (0.9) −0.17 (0.3) −0.14 (0.4) −0.097 (0.5) −0.29 (0.06) −0.21 (0.2) 0.34 (0.03) 0.0067 (1) −0.15 (0.3) −0.28 (0.07) 0.22 (0.2)
MEskyblue −0.32 (0.04) 0.1 (0.5) 0.065 (0.7) 0.062 (0.7) −0.44 (0.003) −0.22 (0.2) 0.046 (0.8) −0.17 (0.3) −0.024 (0.9) 0.051 (0.7) 0.15 (0.3) −0.11 (0.5) −0.28 (0.07) 0.21 (0.2)
MEmidnightblue −0.6 (3e−05) −0.047 (0.8) −0.058 (0.7) 0.0046 (1) −0.56 (1e−04) −0.31 (0.05) 0.14 (0.4) −0.64 (4e−06) −0.54 (2e−04) 0.38 (0.01) 0.068 (0.7) −0.27 (0.08) −0.12 (0.5) 0.23 (0.1)
MEorangered4 −0.51 (5e−04) 0.14 (0.4) 0.11 (0.5) 0.054 (0.7) −0.67 (1e−06) −0.25 (0.1) 0.18 (0.2) −0.54 (2e−04) −0.46 (0.002) 0.27 (0.09) −0.11 (0.5) −0.38 (0.01) −0.033 (0.8) 0.25 (0.1)
MEsteelblue −0.61 (2e−05) −0.33 (0.04) −0.25 (0.1) −0.15 (0.4) −0.27 (0.08) −0.34 (0.03) −0.38 (0.01) −0.55 (1e−04) −0.41 (0.006) 0.29 (0.06) 0.11 (0.5) −0.39 (0.01) −0.19 (0.2) 0.35 (0.02)
MEblue −0.75 (8e−09) −0.16 (0.3) −0.097 (0.5) −0.095 (0.5) −0.6 (2e−05) −0.25 (0.1) 0.29 (0.07) −0.44 (0.003) −0.39 (0.01) 0.48 (0.001) −0.072 (0.7) −0.39 (0.01) −0.21 (0.2) 0.34 (0.03)
MEpaleturquoise −0.87 (7e−14) −0.2 (0.2) −0.21 (0.2) −0.027 (0.9) −0.67 (1e−06) −0.22 (0.2) 0.23 (0.1) −0.6 (3e−05) −0.53 (3e−04) 0.57 (8e−05) −0.056 (0.7) −0.51 (6e−04) −0.096 (0.5) 0.36 (0.02)
FTLD_Status
FTLD_TDP
FTLD_C9orf72
FTLD_GRN
FTLD_MAPT
Onset
Duration
Neurodegen_FCTX
Neurodegen_TCTX
Age
Sex
DoubleN
NeuNP
Sox10P
1
0.5
0
−0.5
−1

c) **Module−trait relationships (k−means) of FTLD3**

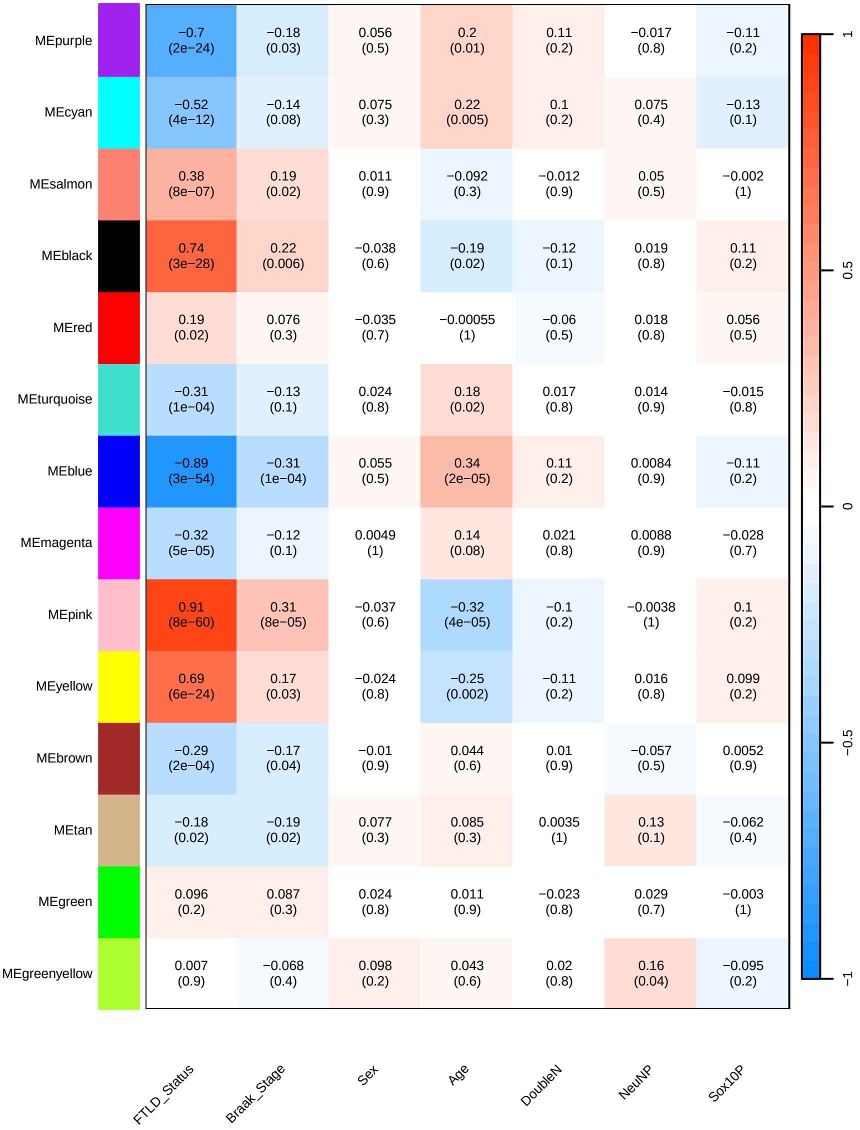

Supplementary figure 4. Module-trait correlations for the FTLD co-methylation networks. a) FTLD1; b) FTLD3; c) FTLD2. The rows represent the co-methylation module eigengene (ME) and its colour, and the columns represent clinical/pathological traits. P values are presented within each cell and the colour scale at the right indicates the strength of the correlation (darker cells depict stronger correlations, with blue representing negative and red positive correlations).

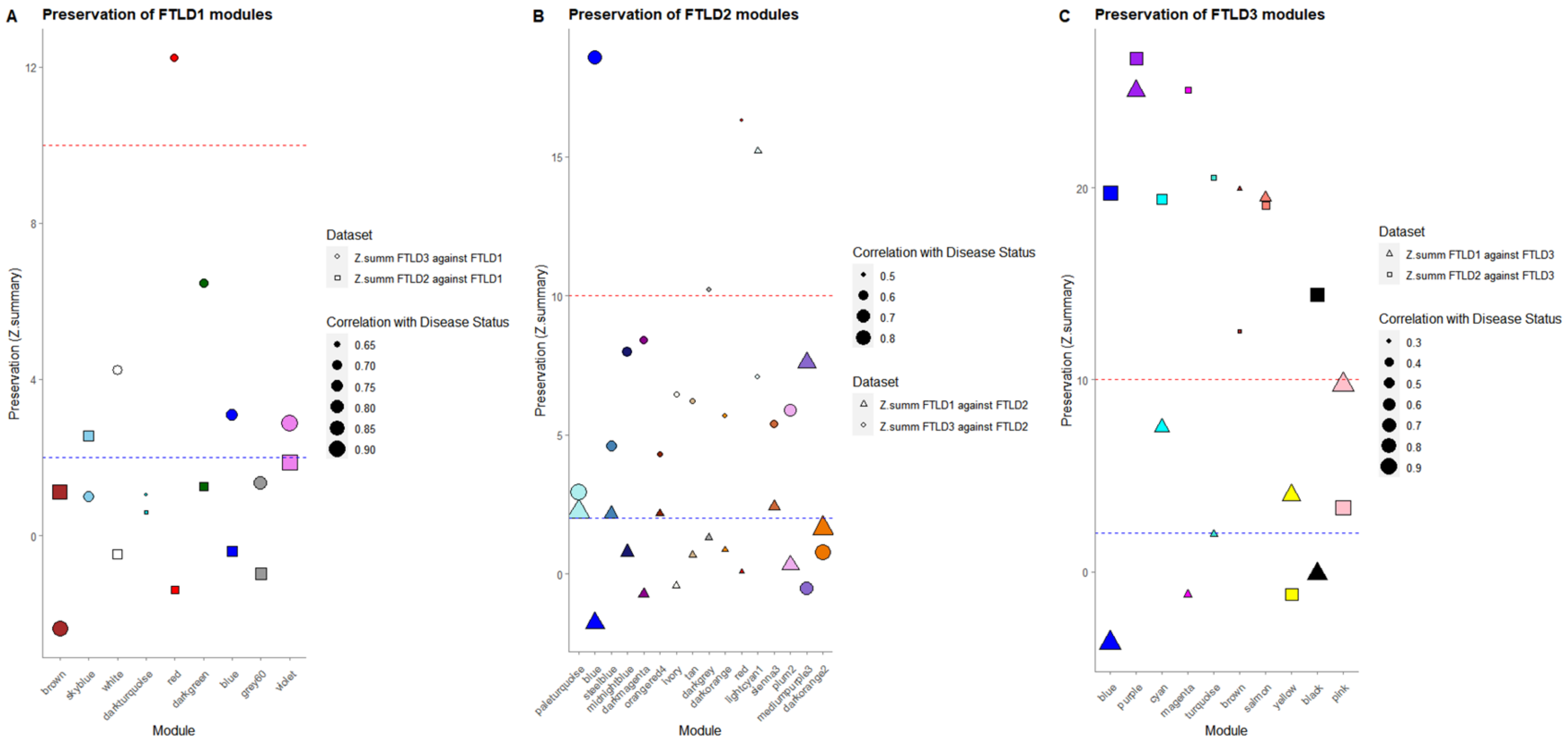

Supplementary figure 5. Preservation analysis Z-summary results for the co-methylation modules associated with FTLD in each individual network. a) Preservation of the FTLD1 network FTLD-associated modules in the FTLD2 (squares) and FTLD3 (circles) data; **b)** Preservation of the FTLD2 network FTLD-associated modules in the FTLD1 (triangles) and FTLD3 (circles) data; **c)** Preservation of the FTLD3 network FTLD-associated modules in the FTLD1 (triangles) and FTLD2 (squares) data. Colour symbols represent co-methylation modules associated with the FTLD status in the corresponding network, and the size of the symbols represents the magnitude of the association. Modules on the left hand side of the plot show the strongest negative associations with the FTLD status, while those on the right show the strongest positive associations with the FTLD status. Y-axis represents the preservation Z.summary. Modules above the red dashed line (Z.summary >10) are said to be highly preserved, modules between the blue and red dashed lines (2< Z.summary <10) are said to be moderately preserved. Modules under the blue dashed line (Z.summary <2) are not preserved.

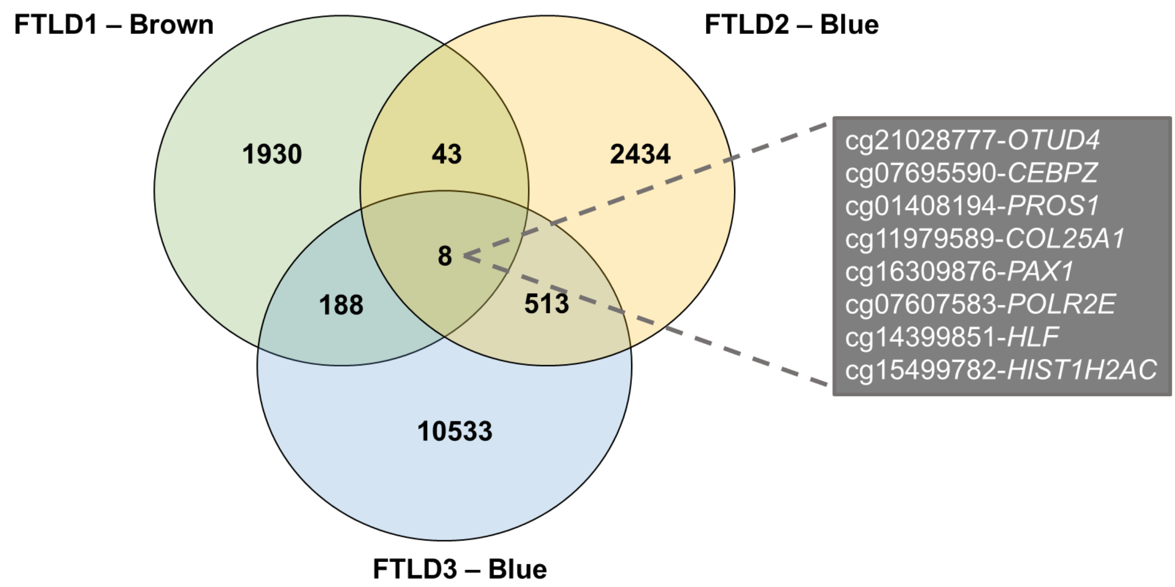

Supplementary figure 6. CpG overlap across the FTLD meta-hit enriched modules. From the three independent co-methylation networks three modules (FTLD1 – Brown, FTLD2 – Blue and FTLD3 – Blue) were enriched for the FTLD top EWAS meta-analysis hits. The eight probes that are present in all three modules are highlighted.

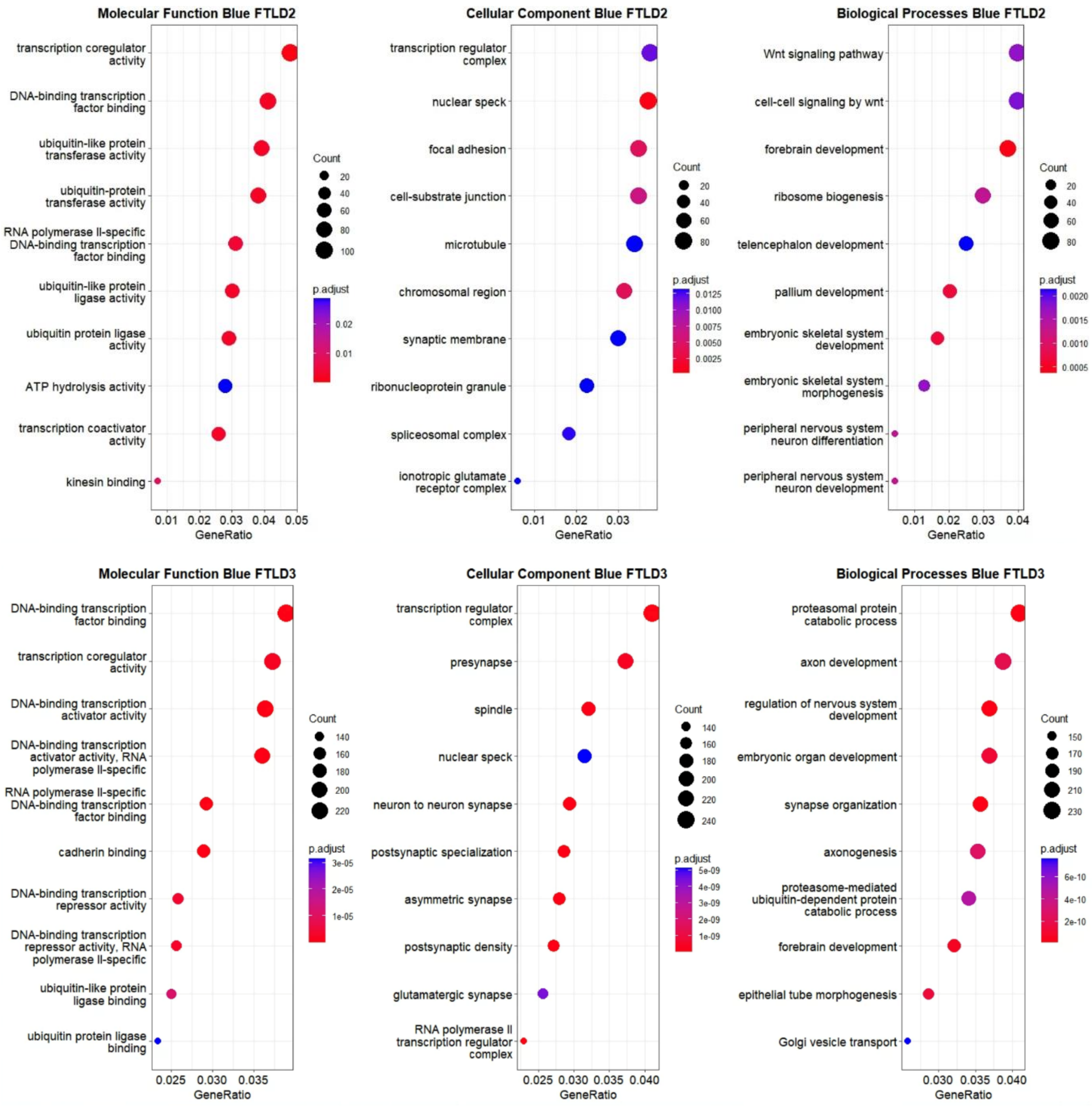

Supplementary figure 7. Functional enrichment for the co-methylation modules enriched for top meta-analysis hits, FTLD2-blue and FTLD3-blue. Note: The FTLD1-brown did not retrieve significant enrichment.

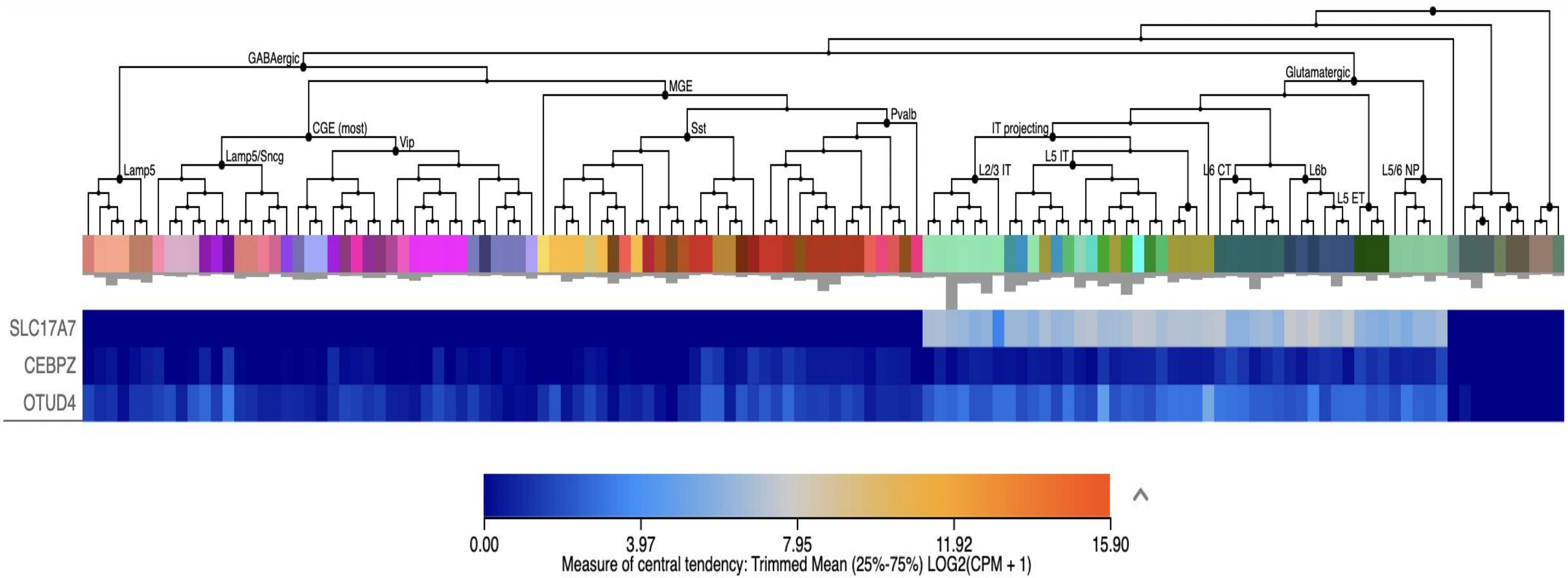

Supplementary figure 8. Single cell expression patterns of a marker of glutamatergic neurons (*SLC17A7*) and the EWAS meta-analysis hits *OTUD4* and *CEBPZ*. Single nuclei RNAseq data from the Allen Brain Map (https://celltypes.brain-map.org/)[Bakken et al., 2021[1]]. © 2016 Allen Institute for Brain Science.

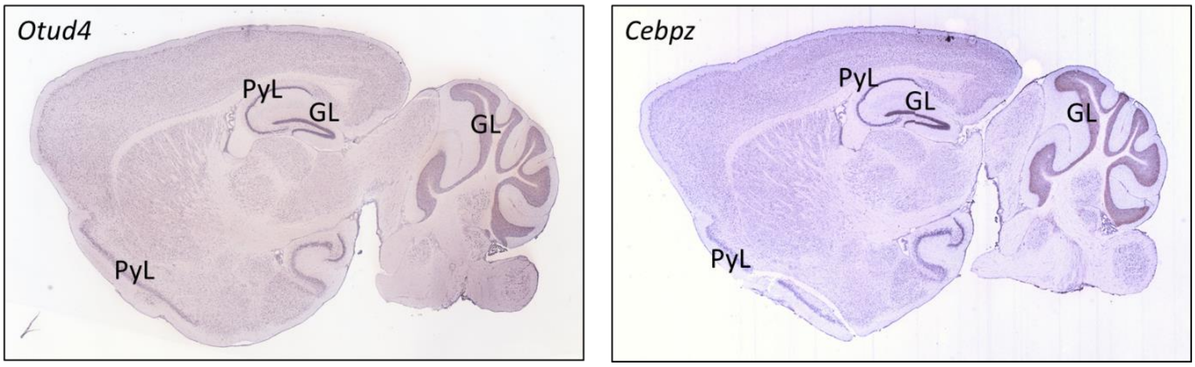

Supplementary figure 9. Expression patterns of EWAS meta-analysis hits *OTUD4* and *CEBPZ* in mice brains. Data from the Allen Brain Atlas (http://mouse.brain-map.org) (Lein et al., 2007[3]). PyL – cortical/hippocampal pyramidal layers; GL – cerebellar/hippocampal granule cell layers. © 2016 Allen Institute for Brain Science

a)

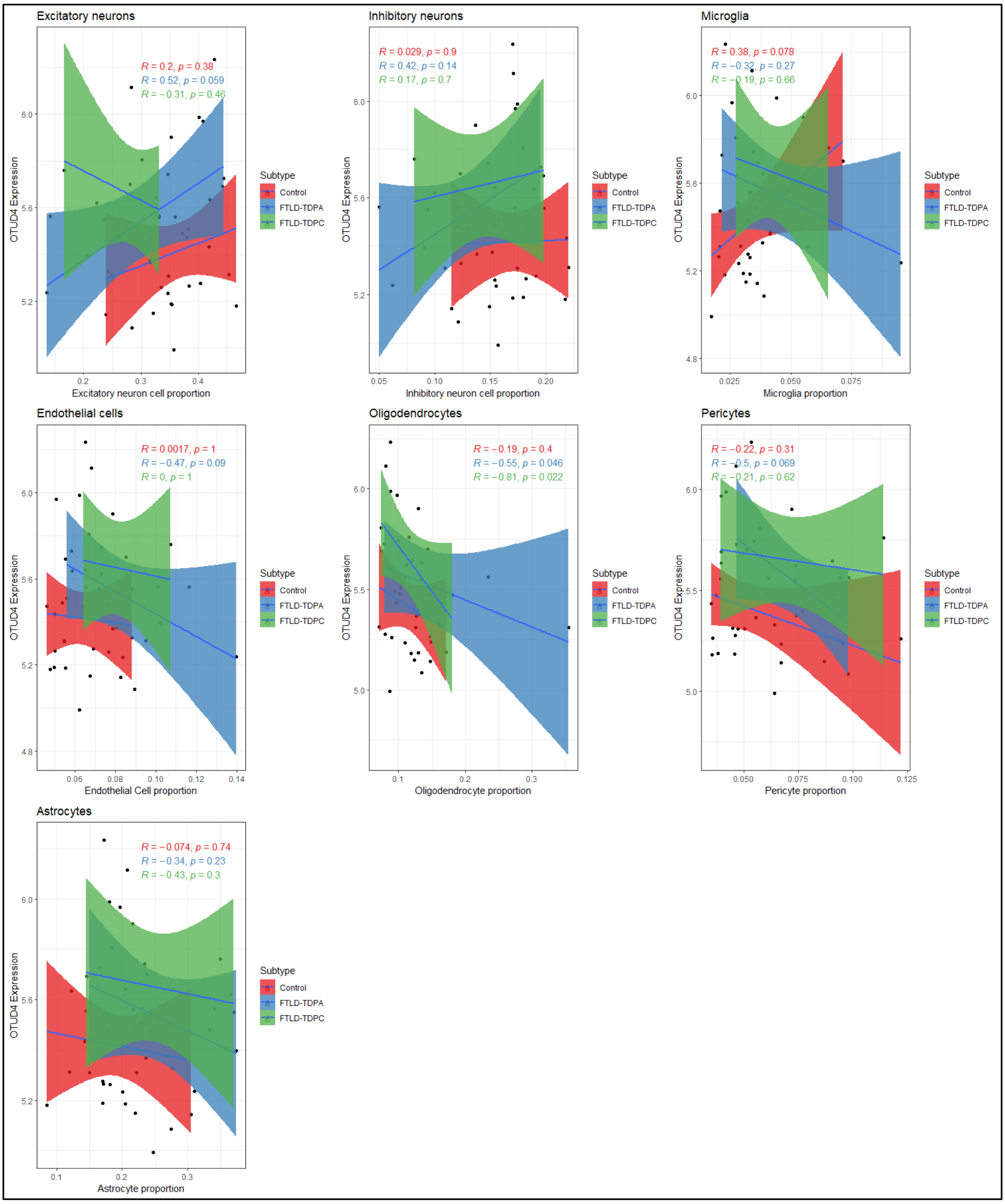
Excitatory neurons
R = 0.2, p = 0.38
R = 0.52, p = 0.059
R = −0.31, p = 0.46
OTUD4 Expression
Excitatory neuron cell proportion
Inhibitory neurons
R = 0.029, p = 0.9
R = 0.42, p = 0.14
R = 0.17, p = 0.7
Inhibitory neuron cell proportion
Microglia
R = 0.38, p = 0.078
R = −0.32, p = 0.27
R = −0.19, p = 0.66
Microglia proportion
Endothelial cells
R = 0.0017, p = 1
R = −0.47, p = 0.09
R = 0, p = 1
Endothelial Cell proportion
Oligodendrocytes
R = −0.19, p = 0.4
R = −0.55, p = 0.046
R = −0.81, p = 0.022
Oligodendrocyte proportion
Pericytes
R = −0.22, p = 0.31
R = −0.5, p = 0.069
R = −0.21, p = 0.62
Pericyte proportion
Astrocytes
R = −0.074, p = 0.74
R = −0.34, p = 0.23
R = −0.43, p = 0.3
Astrocyte proportion
Subtype
Control
FTLD-TDPA
FTLD-TDPC

b)

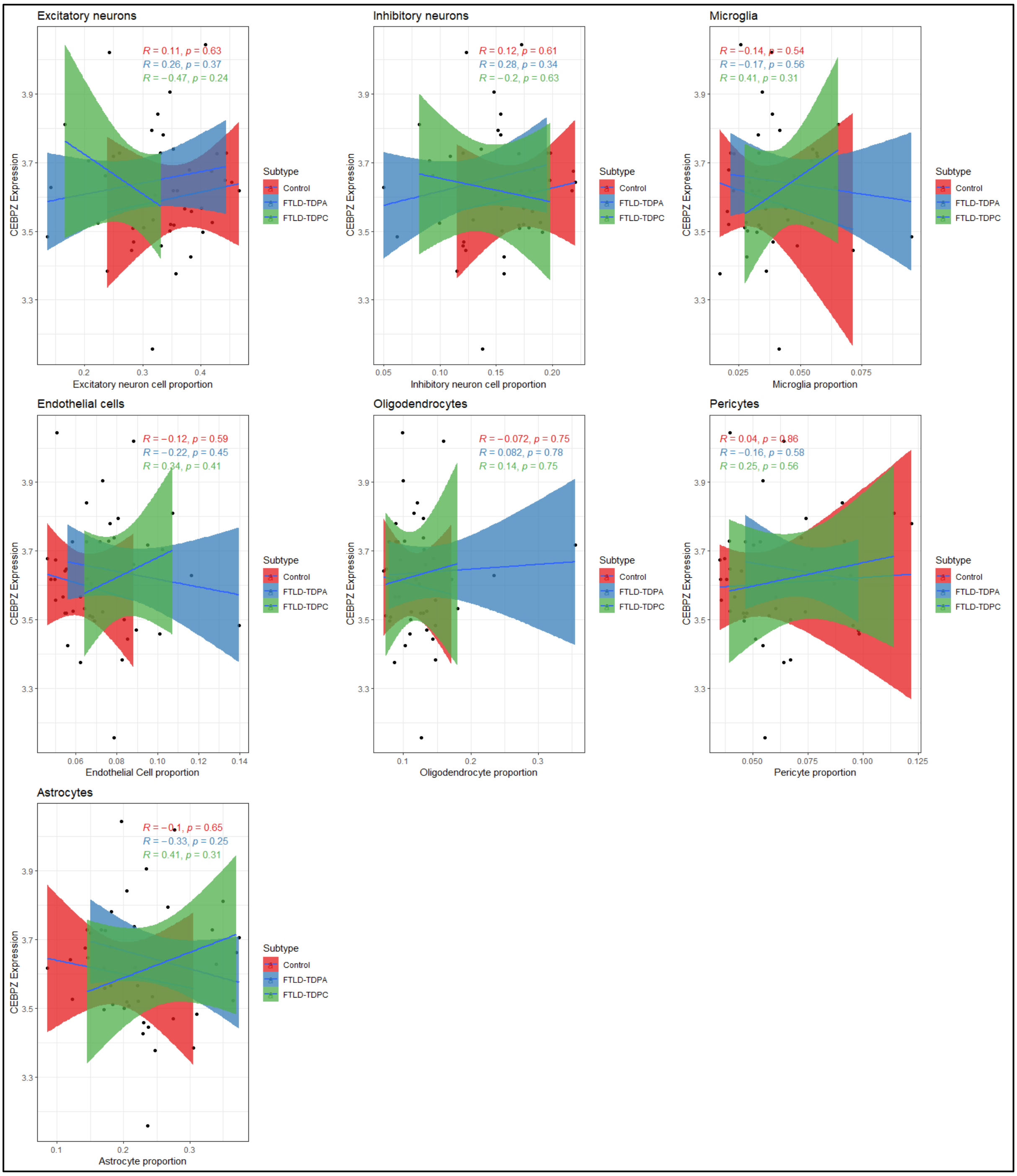

Supplementary figure 10. Relationship between cellular proportions and gene expression levels of meta-hits. a) *OTUD4* gene expression vs cell type proportions; **b)** *CEBPZ* gene expression vs cell type proportions. Expression data from Hasan et al. (2022)[3].

References

1. Bakken TE, Jorstad NL, Hu Q, Lake BB, Tian W, Kalmbach BE, Crow M, Hodge RD, Krienen FM, Sorensen SA, Eggermont J, Yao Z, Aevermann BD, Aldridge AI, Bartlett A, Bertagnolli D, Casper T, Castanon RG, Crichton K, Daigle TL, Dalley R, Dee N, Dembrow N, Diep D, Ding SL, Dong W, Fang R, Fischer S, Goldman M, Goldy J, Graybuck LT, Herb BR, Hou X, Kancherla J, Kroll M, Lathia K, van Lew B, Li YE, Liu CS, Liu H, Lucero JD, Mahurkar A, McMillen D, Miller JA, Moussa M, Nery JR, Nicovich PR, Niu SY, Orvis J, Osteen JK, Owen S, Palmer CR, Pham T, Plongthongkum N, Poirion O, Reed NM, Rimorin C, Rivkin A, Romanow WJ, Sedeño-Cortés AE, Siletti K, Somasundaram S, Sulc J, Tieu M, Torkelson A, Tung H, Wang X, Xie F, Yanny AM, Zhang R, Ament SA, Behrens MM, Bravo HC, Chun J, Dobin A, Gillis J, Hertzano R, Hof PR, Höllt T, Horwitz GD, Keene CD, Kharchenko P v., Ko AL, Lelieveldt BP, Luo C, Mukamel EA, Pinto-Duarte A, Preissl S, Regev A, Ren B, Scheuermann RH, Smith K, Spain WJ, White OR, Koch C, Hawrylycz M, Tasic B, Macosko EZ, McCarroll SA, Ting JT, Zeng H, Zhang K, Feng G, Ecker JR, Linnarsson S, Lein ES (2021) Comparative cellular analysis of motor cortex in human, marmoset and mouse. Nature 2021 598:7879 598:111–119. doi: 10.1038/s41586-021-03465-8

2. Hasan R, Humphrey J, Bettencourt C, Newcombe J, Lashley T, Fratta P, Raj T (2022) Transcriptomic analysis of frontotemporal lobar degeneration with TDP-43 pathology reveals cellular alterations across multiple brain regions. Acta Neuropathol 143:383–401. doi: 10.1007/S00401-021-02399-9/FIGURES/4

3. Lein ES, Hawrylycz MJ, Ao N, Ayres M, Bensinger A, Bernard A, Boe AF, Boguski MS, Brockway KS, Byrnes EJ, Chen L, Chen L, Chen TM, Chin MC, Chong J, Crook BE, Czaplinska A, Dang CN, Datta S, Dee NR, Desaki AL, Desta T, Diep E, Dolbeare TA, Donelan MJ, Dong HW, Dougherty JG, Duncan BJ, Ebbert AJ, Eichele G, Estin LK, Faber C, Facer BA, Fields R, Fischer SR, Fliss TP, Frensley C, Gates SN, Glattfelder KJ, Halverson KR, Hart MR, Hohmann JG, Howell MP, Jeung DP, Johnson RA, Karr PT, Kawal R, Kidney JM, Knapik RH, Kuan CL, Lake JH, Laramee AR, Larsen KD, Lau C, Lemon TA, Liang AJ, Liu Y, Luong LT, Michaels J, Morgan JJ, Morgan RJ, Mortrud MT, Mosqueda NF, Ng LL, Ng R, Orta GJ, Overly CC, Pak TH, Parry SE, Pathak SD, Pearson OC, Puchalski RB, Riley ZL, Rockett HR, Rowland SA, Royall JJ, Ruiz MJ, Sarno NR, Schaffnit K, Shapovalova N v., Sivisay T, Slaughterbeck CR, Smith SC, Smith KA, Smith BI, Sodt AJ, Stewart NN, Stumpf KR, Sunkin SM, Sutram M, Tam A, Teemer CD, Thaller C, Thompson CL, Varnam LR, Visel A, Whitlock RM, Wohnoutka PE, Wolkey CK, Wong VY, Wood M, Yaylaoglu MB, Young RC, Youngstrom BL, Yuan XF, Zhang B, Zwingman TA, Jones AR (2006) Genome-wide atlas of gene expression in the adult mouse brain. Nature 2006 445:7124 445:168–176. doi: 10.1038/nature05453
